# Analysis of the use of monoclonal antibodies in the treatment of Crohn’s disease

**DOI:** 10.1093/abt/tbag006

**Published:** 2026-02-10

**Authors:** Alexander V Blagov, Marina D Sazonova, Anastasia I Ryzhkova, Vasily P Karagodin, Mikhail A Popov, Egor Yu Budnikov, Alessio L Ravani, Alexander N Orekhov, Margarita A Sazonova, Yuri V Arkhipenko

**Affiliations:** Laboratory of Angiopathology, Institute of General Pathology and Pathophysiology, Moscow 125315, Russia; Laboratory of Angiopathology, Institute of General Pathology and Pathophysiology, Moscow 125315, Russia; Laboratory of Angiopathology, Institute of General Pathology and Pathophysiology, Moscow 125315, Russia; Plekhanov Russian University of Economics, Moscow 117997, Russia; Department of Cardiac Surgery, Moscow Regional Research and Clinical Institute ("MONIKI"), Moscow 129110, Russia; Laboratory of Angiopathology, Institute of General Pathology and Pathophysiology, Moscow 125315, Russia; Institute for Atherosclerosis Research, 121609, Moscow, Russia; Institute for Atherosclerosis Research, 121609, Moscow, Russia; Laboratory of Angiopathology, Institute of General Pathology and Pathophysiology, Moscow 125315, Russia; Laboratory of Medical Genetics, Institute of Experimental Cardiology, National Medical Research Center for Cardiology named after academician Y. Chazov of the Ministry of Health of the Russian Federation, Moscow 121552, Russia; Laboratory of Angiopathology, Institute of General Pathology and Pathophysiology, Moscow 125315, Russia; Faculty of Medicine, MSU, Moscow 117192, Russia

**Keywords:** Crohn’s disease, monoclonal antibodies, anti-TNF-α, infliximab, ustekinumab

## Abstract

Crohn’s disease (CD) is a chronic inflammatory bowel disease with increasing global prevalence, significantly impacting patients’ quality of life and healthcare costs. The introduction of monoclonal antibodies has revolutionized CD management, offering targeted therapy against specific inflammatory pathways. This review systematically analyzes the current state of monoclonal antibody therapy, including anti-TNF-α agents (infliximab, adalimumab, certolizumab pegol), anti-integrin antibodies (vedolizumab), and anti-cytokine therapies (ustekinumab, risankizumab). Despite remarkable therapeutic advances, significant limitations persist, including primary non-response (20%–40%), secondary loss of response (13%–20% annually), immunogenicity, safety concerns, and substantial economic burden. We propose evidence-based strategies to address these challenges, including therapeutic drug monitoring, combination therapy, and personalized medicine approaches. Furthermore, we identify promising novel therapeutic targets such as IL-36, IL-17C, SMAD7, TL1A, complement components, and microbiome-related factors. Targeting two or more specific targets simultaneously appears to be a promising direction of research for the development of bi- and polyspecific monoclonal antibodies capable of interfering with multiple pathological pathways in CD. The integration of advanced antibody engineering, personalized medicine, and innovative delivery systems represents the future direction for overcoming current limitations. Achieving sustained remission for all patients through safe, effective, and accessible therapeutic interventions remains the ultimate goal in CD management.

## Teaser

**Statement of Significance**
 

- Despite clinical efficacy and safety, antibodies currently used for the treatment of Crohn’s disease have a number of limitations which can be addressed in two ways.
- The first is to modify treatment approaches and the use of these antibodies.
- The second is aimed at finding new therapeutic targets and developing new monoclonal antibodies.

## Introduction

Crohn’s disease (CD) is a chronic inflammatory bowel disease (IBD) characterized by transmural inflammation that can affect any part of the gastrointestinal tract from the mouth to the anus, with a predilection for the terminal ileum and colon [1]. The disease manifests as a relapsing–remitting condition with periods of active inflammation alternating with remission, often resulting in complications such as strictures, fistulas, and perforations [2]. The global incidence of CD has shown a steady increase over the past several decades, with the highest prevalence rates reported in North America and Europe, ranging from 50 to 200 cases per 100 000 individuals [3]. Emerging data indicate a rising incidence in previously low-prevalence regions, including Asia, South America, and Africa, suggesting a global expansion of the disease burden [4]. This epidemiological shift has been attributed to environmental factors such as westernization of diet, urbanization, improved sanitation paradoxically leading to altered microbiome development, and increased diagnostic awareness [5]. The substantial impact of CD on patients’ quality of life, combined with significant healthcare costs estimated at billions of dollars annually worldwide, underscores the critical need for effective therapeutic interventions [6].

The therapeutic landscape for CD has evolved significantly from conventional treatments to targeted biologic therapies [7]. Traditional treatment approaches include aminosalicylates (5-ASA compounds) for mild disease, corticosteroids for acute flares, and immunosuppressive agents such as azathioprine, 6-mercaptopurine, and methotrexate for maintenance therapy [8]. However, these conventional therapies often provide limited efficacy, carry significant side effect profiles, and fail to address the underlying inflammatory pathways driving the disease [9]. The introduction of biologic therapies, particularly monoclonal antibodies, has revolutionized CD management by offering targeted intervention against specific inflammatory mediators [10]. Anti-tumor necrosis factor-alpha (anti-TNF-α) monoclonal antibodies, including infliximab, adalimumab, and certolizumab pegol, were the first biologics to demonstrate significant efficacy in inducing and maintaining remission in moderate to severe CD [11]. Subsequently, monoclonal antibodies targeting alternative pathways have been developed, including ustekinumab (anti-IL-12/IL-23), vedolizumab (anti-α4β7 integrin), and more recently, risankizumab (anti-IL-23) [12]. These targeted therapies have shown superior efficacy compared to conventional treatments, with improved rates of clinical remission, mucosal healing, and reduced need for surgery, establishing monoclonal antibodies as cornerstones of modern CD therapy [13].

Despite the remarkable advances in monoclonal antibody (mAb) therapy for CD, several significant limitations persist that warrant comprehensive analysis and innovative solutions [14]. The objectives of this review are threefold: first, to conduct a systematic analysis of existing limitations in mAb therapy for CD, including primary non-response affecting 20%–40% of patients, secondary loss of response observed in 13%–20% of patients annually, immunogenicity leading to treatment failure, significant adverse events including increased infection risk and potential malignancy, and the substantial economic burden limiting patient access [15]. Second, to propose evidence-based strategies for addressing these limitations, including optimization of dosing regimens, combination therapy approaches, therapeutic drug monitoring, immunogenicity mitigation strategies, and patient stratification methods for personalized treatment selection [16]. Third, to identify and evaluate potentially novel therapeutic targets for future mAb development in CD, including emerging cytokine pathways (IL-36, IL-17, SMAD7), cell adhesion molecules, complement system components, and microbiome-related targets, which may offer improved efficacy, reduced side effects, and solutions for treatment-refractory patients, ultimately advancing the field toward more precise and effective therapeutic interventions [17].

## Current state of approved and emerging monoclonal antibody in CD therapy

### Anti-TNF-α monoclonal antibodies

#### Infliximab

Infliximab, a chimeric mAb targeting tumor necrosis factor-alpha, represents the pioneering biologic therapy in CD management [18]. The landmark ACCENT I trial demonstrated infliximab’s efficacy in inducing clinical remission in 58% of patients at week 10, with maintenance of remission achieved in 39% of patients at week 54 compared to 21% with placebo [11]. Subsequent real-world studies have confirmed these findings, showing clinical response rates of 60%–70% in induction therapy and remission maintenance in ~40%–50% of patients over 1 year [19]. The SONIC trial further established infliximab’s superior efficacy when combined with azathioprine, demonstrating corticosteroid-free remission in 56.8% of combination therapy patients versus 44.4% with infliximab monotherapy [20]. However, infliximab therapy is associated with significant immunogenicity, with anti-drug antibodies developing in 10%–61% of patients, leading to treatment failure and increased infusion reactions [21].

#### Adalimumab

Adalimumab, a fully human anti-TNF-α mAb, offers the advantage of subcutaneous administration and potentially reduced immunogenicity compared to infliximab [22]. The CLASSIC I trial demonstrated clinical remission rates of 36% at week 4 and 59% at week 26 in patients receiving adalimumab induction therapy [23]. The CHARM study revealed that adalimumab maintenance therapy achieved clinical remission in 40% of patients at week 26 and 36% at week 56, significantly superior to placebo [24]. Meta-analyses indicate that adalimumab demonstrates comparable efficacy to infliximab, with clinical response rates of 58%–67% for induction and 47%–52% for maintenance therapy [25]. The development of anti-adalimumab antibodies occurs in ~2.6%–26.5% of patients, generally lower than infliximab, though this remains a significant concern for long-term efficacy [26].

#### Certolizumab pegol

Certolizumab pegol, a pegylated anti-TNF-α antibody fragment, provides unique pharmacokinetic properties with prolonged half-life and minimal placental transfer [27]. The PRECISE 1 trial demonstrated clinical response rates of 64.1% at week 6 and 62.4% at week 26 in patients receiving certolizumab pegol 400 mg [28]. The PRECISE 2 maintenance study showed sustained clinical remission in 48% of responders at week 26 compared to 29% with placebo [29]. Notably, certolizumab pegol has demonstrated particular efficacy in patients with prior anti-TNF therapy failure, with response rates of 43.8% in the WELCOME study [30]. The immunogenicity profile appears favorable, with anti-drug antibodies detected in ~8%–12% of patients, though longer-term data are still accumulating [31].

### Anti-integrin monoclonal antibodies

#### Vedolizumab

Vedolizumab represents a paradigm shift toward gut-selective therapy, targeting α4β7 integrin to prevent lymphocyte migration into gastrointestinal tissues [32]. The GEMINI 2 trial demonstrated clinical remission rates of 14.5% at week 6 and 39% at week 52 in CD patients receiving vedolizumab [13]. While the induction response appears slower compared to anti-TNF therapies, vedolizumab shows particular efficacy in anti-TNF-experienced patients, with clinical response rates of 31.4% at week 10 [33]. The VERSIFY study confirmed vedolizumab’s effectiveness in real-world settings, showing clinical remission in 47.7% of patients at week 52 [34]. The gut-selective mechanism results in an excellent safety profile with minimal systemic immunosuppression, and anti-vedolizumab antibodies are detected in less than 4% of patients [35].

### Anti-IL-12/IL-23 monoclonal antibodies

#### Ustekinumab

Ustekinumab targets the p40 subunit common to both IL-12 and IL-23, disrupting key inflammatory pathways in CD [36]. The UNITI-1 trial in anti-TNF-naive patients demonstrated clinical response rates of 51.7% at week 6, while UNITI-2 in anti-TNF-experienced patients showed response rates of 40.2% [12]. The IM-UNITI maintenance study revealed clinical remission in 53.1% of patients at week 44 with 90 mg dosing every 8 weeks [37]. Ustekinumab demonstrates consistent efficacy across different patient populations, including those with prior biologic failure, with sustained clinical benefits observed in long-term extension studies [38]. The immunogenicity profile is favorable, with anti-ustekinumab antibodies detected in ~2.3%–5.1% of patients [39].

#### Risankizumab

Risankizumab, a selective IL-23 inhibitor targeting the p19 subunit, represents the latest advancement in cytokine-targeted therapy for CD [40]. The ADVANCE trial demonstrated superior clinical remission rates of 42% at week 12 compared to 32% with placebo [41]. The FORTIFY maintenance study showed clinical remission in 45% of patients at week 52 with 180 mg dosing every 8 weeks [42]. Added 3-year extension data from FORTIFY showing sustained remission in 68% of continuous responders and favorable safety profile with no new safety signals (presented at DDW 2024) [43]. Risankizumab has shown particular promise in patients with prior biologic failure, with response rates of 38% in the MOTIVATE trial [44]. The selective IL-23 targeting may offer advantages over broader IL-12/IL-23 inhibition, with preliminary safety data suggesting a favorable profile and low immunogenicity rates of ~1%–3% [45].

### Emerging monoclonal antibodies

Several novel monoclonal antibodies are currently in clinical development for CD. Mirikizumab, another IL-23 inhibitor, has demonstrated promising results in phase 3 trials with clinical remission rates of 24.2% at week 12 [46]. Brazikumab, targeting IL-23 receptor, showed clinical response rates of 49.2% in phase 2 studies [47]. Etrolizumab, an anti-β7 integrin antibody, demonstrated clinical remission in 21.6% of patients at week 14 in the BERGAMOT trial [48].

Tables 1 and 2 summarizes the results of clinical trials of monoclonal antibodies for the treatment of CD.

**Table 1:** Clinical efficacy of monoclonal antibodies in Crohn’s disease with patients baseline characteristics.

Monoclonal antibody	Target	Key trial	Patient population	Concomitant therapy	Induction response rate	Maintenance remission rate	Placebo response/remission	Anti-drug antibodies	Key advantages
Infliximab	TNF-α	ACCENT I	Bio-naïve and bio-experienced mixed	Combination with AZA in SONIC (56.8% vs. 44.4% monotherapy)	58%–70%	39%–50%	21% remission at week 54	10%–61%	Established efficacy, IV administration
Adalimumab	TNF-α	CLASSIC I/CHARM	Majority bio-naïve (CLASSIC I: 100% bio-naïve)	Monotherapy primarily, some with immunomodulators	58%–67%	36%–52%	17% remission at week 26 (CHARM)	2.6%–26.5%	Subcutaneous dosing, lower immunogenicity
Certolizumab pegol	TNF-α	PRECISE 1/2, WELCOME	WELCOME: 100% prior anti-TNF failure	Monotherapy primarily	62%–64%	48%	29% remission at week 26 (PRECISE 2)	8%–12%	Pregnancy safety, anti-TNF failure efficacy
Vedolizumab	α4β7 integrin	GEMINI 2	Mixed: 47% prior anti-TNF exposure	Monotherapy or with stable immunomodulators	31%–47%	39%–48%	21.6% remission at week 52	<4%	Gut-selective, excellent safety profile
Ustekinumab	IL-12/IL-23	UNITI-1/2, IM-UNITI	UNITI-1: bio-naïve; UNITI-2: prior anti-TNF failure	Monotherapy primarily	40%–52%	53%	27.5% remission at week 44	2.3%–5.1%	Broad efficacy, biologic failure response
Risankizumab	IL-23	ADVANCE, MOTIVATE, FORTIFY	ADVANCE: 59% bio-naïve; MOTIVATE: 100% prior biologic failure	Monotherapy primarily	38%–42%	45%	25% remission at week 12 (ADVANCE)	1%–3%	Selective targeting, low immunogenicity

**Table 2:** Clinical characteristics, safety profiles, and positioning of monoclonal antibodies in Crohn’s disease.

Antibody class	Representative agents	Induction response rate	Maintenance remission rate (52 weeks)	Safety profile	Preferred patient populations	Typical clinical positioning
Anti-TNF-α	Infliximab, Adalimumab, Certolizumab pegol	58%–70%	36%–50%	Serious infections: 4–6 per 100 PY; Tuberculosis reactivation: 0.2%–0.5%; Infusion/injection reactions: 5%–10%; Malignancy risk: OR 1.4 (lymphoma)	Moderate–severe luminal disease; Perianal fistulizing disease; Patients requiring rapid symptom control; Inflammatory non-stricturing phenotype	First-line biologic in most guidelines; Combination with immunomodulators increases efficacy
Anti-α4β7 integrin	Vedolizumab	31%–47%	39%–48%	Serious infections: 2.4 per 100 PY; Minimal systemic immunosuppression; Infusion reactions: 4%; PML: extremely rare (<0.01%)	Anti-TNF-experienced patients; Patients with infection concerns; Elderly patients; Concomitant extraintestinal manifestations not requiring treatment	Second-line after anti-TNF failure; First-line in patients with contraindications to anti-TNF
Anti-IL-12/IL-23 (p40)	Ustekinumab	40%–52%	53%	Serious infections: 3.0 per 100 PY; MACE: 0.5 per 100 PY; Low immunogenicity; Well-tolerated SC administration	Broad applicability across phenotypes; Biologic-experienced patients; Patients prioritizing convenient dosing	Second-line after anti-TNF failure; Emerging as first-line option
Anti-IL-23 (p19)	Risankizumab, Mirikizumab	38%–45%	45%–52%	Serious infections: 2.8 per 100 PY; Hepatic events: monitor LFTs; Low immunogenicity (1%–3%); Excellent drug survival	Inflammatory luminal disease; Patients prioritizing long-term durability; Failed multiple prior biologics; Infection-prone patients	Second-line after anti-TNF failure; Consideration for first-line in select patients (elderly, infection concerns, preference for low immunogenicity)

PY, patient-years; SC, subcutaneous; MACE, major adverse cardiovascular events; PML, progressive multifocal leukoencephalopathy; LFTs, liver function tests; OR, odds ratio.

**Figure 1:**
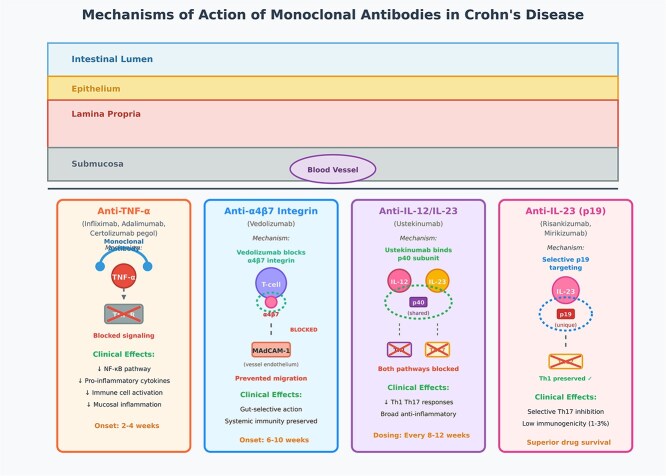
Mechanisms of action of monoclonal antibodies in Crohn’s disease treatment.

Figure 1 shows mechanisms of action of monoclonal antibodies in CD treatment.

## Current limitations of approved monoclonal antibodies for CD treatment and solutions

### Primary non-response

Primary non-response represents a significant challenge in mAb therapy for CD, affecting ~20%–40% of patients across different therapeutic agents [15]. This phenomenon occurs when patients fail to achieve clinical response despite adequate drug exposure during induction therapy [49]. The mechanisms underlying primary non-response are multifactorial and include genetic polymorphisms affecting drug metabolism, variations in target expression, disease heterogeneity, and individual immune system characteristics [50].

Several strategies have been developed to address primary non-response. Pharmacogenomic testing can identify patients with genetic variants affecting drug metabolism, particularly for anti-TNF agents where polymorphisms in TNF-α promoter regions and HLA alleles influence treatment response [51]. Dose optimization based on therapeutic drug monitoring during induction has shown promise in improving initial response rates [52]. Additionally, biomarker-guided therapy selection, including assessment of mucosal TNF-α expression, serum cytokine profiles, and histologic features, can help identify patients more likely to respond to specific therapeutic targets [53].

### Secondary loss of response

Secondary loss of response affects 13%–20% of patients annually and represents a major clinical challenge in maintaining long-term remission [14]. This phenomenon typically occurs after an initial therapeutic response and can result from multiple mechanisms including immunogenicity, disease progression, development of neutralizing antibodies, and changes in drug pharmacokinetics [54].

Therapeutic drug monitoring has emerged as a crucial tool for managing secondary loss of response [16]. Proactive monitoring of drug levels and anti-drug antibodies allows for timely dose optimization before clinical deterioration occurs [55]. Meta-analyses demonstrate that proactive monitoring compared to reactive monitoring results in improved clinical outcomes, reduced immunogenicity, and better long-term treatment persistence [56]. Combination immunosuppressive therapy with methotrexate or azathioprine can prevent anti-drug antibody formation and maintain therapeutic drug levels, particularly for anti-TNF agents [20].

### Immunogenicity and treatment failure

Immunogenicity remains a significant limitation across all mAb therapies, though rates vary considerably between agents [21]. The development of anti-drug antibodies leads to accelerated drug clearance, reduced efficacy, and increased risk of infusion or injection site reactions [57]. Factors influencing immunogenicity include drug structure (chimeric vs. humanized vs. fully human), dosing frequency, concomitant immunosuppression, and individual patient characteristics [58].

Several approaches have been developed to mitigate immunogenicity. Combination therapy with immunosuppressive agents significantly reduces antibody formation, with the SONIC trial demonstrating superior outcomes for infliximab plus azathioprine compared to monotherapy [20]. Prophylactic treatment with corticosteroids or antihistamines can reduce acute infusion reactions [59]. Novel antibody engineering approaches, including Fc modifications and alternative dosing regimens, are being investigated to reduce immunogenic potential while maintaining therapeutic efficacy [60].

### Safety concerns and adverse events

Monoclonal antibody therapies carry significant safety risks that limit their use in certain patient populations [61]. Anti-TNF agents increase the risk of serious infections, including opportunistic infections, reactivation of latent tuberculosis, and hepatitis B virus reactivation [62]. There are also concerns about increased malignancy risk, particularly lymphoma, and rare but serious adverse events such as demyelinating disease and heart failure [63].

Risk mitigation strategies include comprehensive screening protocols before treatment initiation, including testing for latent tuberculosis, hepatitis B and C, and assessment of cardiovascular status [64]. Regular monitoring during therapy with complete blood counts, liver function tests, and clinical assessment for signs of infection is essential [65]. Patient education about infection prevention and early recognition of symptoms is crucial for optimal safety outcomes [66].

Monoclonal antibody therapies demonstrate class-specific safety profiles reflecting their distinct mechanisms of action, with critical implications for patient selection and risk management [57].

Anti-TNF agents carry the highest infection risk among biologics due to systemic TNF-α blockade, which impairs granuloma formation and innate immunity. Meta-analysis of 22 randomized trials (*n* = 8905) by Lichtenstein et al. (2012) demonstrated serious infection rates of 4.6 per 100 patient-years, significantly elevated versus placebo (OR 2.05, 95% CI 1.10–3.85) [61]. Opportunistic infections include tuberculosis reactivation (incidence 0.2%–0.5% with screening protocols), invasive fungal infections (0.3%–0.8%), and hepatitis B reactivation (10%–25% in HBsAg-positive patients without prophylaxis) [62]. Malignancy concerns include increased lymphoma risk (OR 1.4, 95% CI 1.1–1.8), particularly hepatosplenic T-cell lymphoma in young males receiving combination therapy, and possible melanoma risk (standardized incidence ratio 1.4–1.5) [63]. Demyelinating disorders occur rarely (incidence <0.1%) but mandate discontinuation [67]. The TREAT registry with >6000 patient-years follow-up confirmed dose-dependent infection risk, with intensified dosing increasing serious infection rates by 30%–40% [61].

Vedolizumab demonstrates superior safety through gut-selective α4β7 integrin blockade that prevents lymphocyte trafficking specifically to intestinal tissue while preserving systemic immunity. The GEMINI long-term safety study (*n* = 2830, median 2.3 years follow-up) reported serious infection rates of only 2.4 per 100 patient-years, significantly lower than anti-TNF agents (adjusted HR 0.52, 95% CI 0.38–0.71) [35]. Critically, no increased risk of opportunistic infections, tuberculosis, or progressive multifocal leukoencephalopathy (PML) has been observed, contrasting with natalizumab’s pan-integrin blockade which carries 1:1000 PML risk [68]. Malignancy rates match background population (standardized incidence ratio 0.98, 95% CI 0.72–1.31) [69]. The mechanism-based safety advantage makes vedolizumab particularly suitable for elderly patients (>65 years), those with prior serious infections, chronic viral hepatitis carriers, latent tuberculosis with treatment concerns, and patients with multiple comorbidities requiring polypharmacy [70].

Anti-IL-12/IL-23 and anti-IL-23 agents demonstrate intermediate safety profiles. Ustekinumab meta-analysis (9 trials, *n* = 4135) showed serious infection rates of 3.0 per 100 patient-years, lower than anti-TNF (*P* = .04) but higher than vedolizumab [71]. The IL-23 pathway’s role in mucosal barrier defense explains maintained protection against bacterial infections while reducing inflammatory pathology. Risankizumab demonstrates even more favorable safety with 2.8 serious infections per 100 patient-years and low opportunistic infection incidence (<0.5%) [42]. Theoretical concerns about impaired immunity to extracellular bacteria and fungi have not materialized in clinical practice. A potential signal for major adverse cardiovascular events with ustekinumab (incidence 0.5 per 100 patient-years) requires ongoing surveillance, particularly in patients with cardiovascular risk factors [72]. Hepatic enzyme elevations occur in 8%–12% with IL-23 inhibitors, usually transient and asymptomatic, but mandate monitoring [41].

Risk mitigation strategies include comprehensive pre-treatment screening (tuberculosis, hepatitis B/C, HIV, complete blood count, liver function), age-appropriate vaccinations (pneumococcal, influenza, hepatitis B, herpes zoster if >50 years with inactivated vaccine) administered ≥2–4 weeks before therapy initiation, regular monitoring (complete blood count and liver function every 3–6 months), patient education emphasizing early recognition of infection symptoms, and dose adjustment or temporary discontinuation during active infections [64].

### Economic burden and access limitations

The substantial cost of mAb therapies represents a significant barrier to patient access and healthcare system sustainability [6]. Annual treatment costs can exceed $30 000–50 000 per patient, creating disparities in access based on insurance coverage and geographic location [73]. This economic burden limits the ability to implement optimal treatment strategies and may delay appropriate therapy initiation [74].

Addressing economic barriers requires multifaceted approaches including value-based care models, biosimilar development to reduce costs, patient assistance programs, and healthcare policy reforms [75]. Biosimilar versions of infliximab and adalimumab have demonstrated comparable efficacy and safety while reducing costs by 20%–30% [76]. Pharmacoeconomic analyses support the long-term cost-effectiveness of biologic therapy through reduced hospitalizations, surgery rates, and disability, though upfront costs remain prohibitive for many patients [77].

#### Biosimilar impact on cost and access

Biosimilar introduction has significantly impacted the economic landscape. The NOR-SWITCH trial demonstrated non-inferiority of CT-P13 (infliximab biosimilar) to originator infliximab, with comparable efficacy (29.6% vs. 30.0% disease worsening, 95% CI −7.7 to 5.8) and safety over 52 weeks [76]. Real-world implementation in Norway achieved 34% cost reduction, translating to €11.7 million annual savings for IBD treatment alone [78]. Denmark’s mandatory switching program (2015–2016) achieved 69% cost reduction for infliximab and demonstrated sustained effectiveness with 89% continuation rates at 1 year [79].

US market dynamics show more modest impact, with biosimilar uptake reaching only 45% by 2023 despite FDA approval of multiple biosimilars (CT-P13, SB2, PF-06438179 for infliximab; ABP 501, SB5, GP2017 for adalimumab) [80]. Average wholesale price reductions of 15%–35% versus originators remain below European reductions of 30%–70%, attributed to complex rebate systems and limited interchangeability designation [81]. A 2023 IQVIA analysis projects potential US savings of $6.8 billion over 5 years with increased biosimilar utilization [82].

#### Regional variations in access

High-income countries (North America, Western Europe, Australia): Universal or near-universal biologic access through insurance/national health systems, though prior authorization requirements cause treatment delays averaging 8–12 weeks in US commercial insurance [83]. Cost-sharing responsibilities create patient-level barriers, with out-of-pocket costs of $3000–$8000 annually leading to 15%–25% non-adherence rates [84].

Middle-income countries (Eastern Europe, Latin America, parts of Asia): Heterogeneous access with public-private divide. Brazil’s unified health system provides biologics but faces 6–12 months waiting lists affecting 40% of eligible patients [85]. Mexico’s biosimilar introduction (2016–2018) increased treatment access by 180% with infliximab biosimilar costs at $8500 per patient-year versus $24 000 for originator [86]. Poland’s national program covers biologics for 80% of eligible patients, with biosimilar utilization reaching 75% [87].

Low-income countries (sub-Saharan Africa, South Asia): Severely limited access with <5% of eligible patients receiving biologic therapy in most regions [88]. India’s domestic biosimilar production (BOW015 infliximab, Exemptia adalimumab) achieves costs of $3000–$5000 per patient-year, enabling treatment for middle-class patients but remaining unaffordable for majority populations [89]. Public sector provision virtually absent in most countries.

#### Cost-effectiveness evidence

Recent pharmacoeconomic analyses support biologic therapy cost-effectiveness when considering indirect costs. The UK NICE evaluation (2023 update) found incremental cost-effectiveness ratios of £18 400–£24 600 per quality-adjusted life year (QALY) for anti-TNF therapy versus conventional treatment, well below £30 000 willingness-to-pay threshold [90]. Key drivers include reduced hospitalizations (3.2 vs. 1.4 per patient-year, saving £8400 annually), decreased surgery rates (11% vs. 23% at 5 years, saving £12 000 per avoided surgery), and improved work productivity (65% vs. 45% full-time employment, worth £9200 annually) [77].

IL-23 inhibitors demonstrate favorable cost-effectiveness versus anti-TNF agents when modeling long-term drug survival. Canadian analysis of risankizumab versus adalimumab yielded incremental cost-effectiveness ratio of CAD $32 400 per QALY, driven by 78% versus 65% drug persistence at 3 years reducing treatment switching costs [91]. However, upfront costs remain prohibitive without biosimilar alternatives.

### Practical considerations in biologic therapy selection

Beyond efficacy and safety, practical factors significantly influence treatment outcomes and patient satisfaction, warranting integration into shared decision-making processes [92].

#### Administration routes and patient preferences

Intravenous administration (infliximab, vedolizumab) requires infusion center visits every 4–8 weeks, with 2–3 h infusion times including monitoring periods. Patient surveys indicate 62% prefer home-based administration when given choice, though 38% value supervised infusion for immediate reaction management and healthcare team interaction [93]. Infusion centers enable concurrent laboratory monitoring, therapeutic drug monitoring, and clinical assessments, potentially improving treatment optimization.

Subcutaneous administration (adalimumab, certolizumab, ustekinumab, risankizumab) enables home self-injection, with 85% of patients successfully self-administering after training [94]. Patient preference studies demonstrate higher treatment satisfaction scores with subcutaneous therapy (7.8 vs. 6.9 on 10-point scale, *P* < .001), driven by convenience, reduced time burden (15 min vs. 4 h including travel), and lifestyle flexibility [95]. However, 12%–15% of patients experience injection site reactions, and some report anxiety about self-administration technique [24].

Extended dosing intervals (ustekinumab every 8–12 weeks, risankizumab every 8 weeks) after induction significantly improve convenience compared to every-2-week or every-4-week regimens, with adherence rates of 89% versus 78%, respectively (*P* = .002) [38].

#### Adherence patterns and interventions

Medication non-adherence affects 25%–40% of IBD patients receiving biologic therapy, increasing relapse risk 3.2-fold (95% CI 2.1–4.8) [96]. Primary drivers include cost concerns (affecting 45% of non-adherent patients), forgetfulness (32%), needle phobia (18% for subcutaneous agents), time constraints for infusions (28%), and perceived inefficacy or side effects (24%) [97].

Evidence-based adherence interventions include:

- Patient education programs: Structured education about disease mechanisms, treatment goals, and proper administration techniques improves adherence by 18%–25% [98].
- Reminder systems: Automated text message or app-based reminders increase on-time administration from 72% to 86% [99].
- Nurse-led telephone support: Monthly check-in calls addressing concerns and troubleshooting improve persistence by 22% [100].
- Shared decision-making: Involving patients in treatment selection increases commitment, with adherence rates of 84% when preference-concordant versus 68% when preference-discordant [101].
- Financial assistance programs: Co-pay assistance and patient assistance programs reduce non-adherence due to cost from 45% to 12% [102].

#### Impact on treatment outcomes

Adherence strongly predicts outcomes, with >80% medication possession ratio associated with clinical remission rates of 58% versus 34% with <80% adherence (*P* < .001) [103]. Each 10% decrease in adherence increases hospitalization risk by 15% (HR 1.15, 95% CI 1.08–1.23) and surgery risk by 12% (HR 1.12, 95% CI 1.04–1.21) [104]. Patient-centered approaches addressing practical barriers and preferences optimize both adherence and clinical outcomes.

Current limitations of monoclonal antibodies for CD treatment and their solutions are summarized in Table 3.

**Table 3:** Limitations of monoclonal antibody therapy for Crohn’s disease: Clinical impact, solutions, and evidence base.

Limitation	Prevalence	Clinical impact	Current standard approaches	Innovative/emerging strategies	Evidence level and key references
Primary non-response	20%–40% across agents	Treatment delay (median 12–16 weeks lost); Disease progression during ineffective therapy; Increased risk of complications (OR 2.1 for surgery); Healthcare costs: additional $15 000–25 000 per failed trial	Pharmacogenomic testing for TNF polymorphisms; Biomarker-guided selection (fecal calprotectin, CRP); Dose optimization during induction (e.g., accelerated infliximab dosing)	Machine learning algorithms predicting response (AUC 0.78–0.82); Multi-omics profiling (genomics + proteomics + microbiome); Point-of-care mucosal TNF-α measurement; Adaptive dosing based on early pharmacokinetic sampling	**High evidence**: RCTs for dose optimization [52]; Meta-analysis of biomarkers [53]. **Moderate evidence**: Pharmacogenomics [51]; Machine learning [105]
Secondary loss of response	13%–20% annually; Cumulative 5-year rate: 50%–60%	Clinical relapse requiring hospitalization (15%–20%); Need for dose escalation (30%–40%); Treatment switching costs: $8000–12 000; Surgery rates increase by 8%–10%	Therapeutic drug monitoring (TDM); Proactive TDM superior to reactive (remission: 55% vs. 38%); Combination therapy with immunomodulators; Dose intensification protocols	Dashboard-based TDM with automated dosing recommendations; Prophylactic switching before antibody formation; Novel immunomodulators (e.g., JAK inhibitors) to prevent immunogenicity; Extended interval dosing after deep remission	**High evidence**: Multiple RCTs and meta-analyses for proactive TDM [16, 56]. **Moderate evidence**: Prophylactic switching [106]
Immunogenicity	2%–61% (varies by agent): Infliximab 10%–61%; Adalimumab 2.6%–26.5%; Vedolizumab <4%; Risankizumab 1%–3%	Treatment failure in 30%–40% with high-titer antibodies; Infusion reactions (4%–8%); Accelerated drug clearance (half-life reduced by 40%–60%); Limited re-treatment options	Concomitant immunosuppression (reduces ADA by 50%–70%); Scheduled maintenance dosing; Prophylactic corticosteroids/antihistamines for infusions; Early dose intensification	Fc-modified antibodies with reduced immunogenicity; PEGylation strategies; Induction of immune tolerance protocols; Transient immunosuppression during induction only; Next-generation fully human/humanized constructs	**High evidence**: SONIC trial for combination therapy [20]; Meta-analyses [57]. **Moderate evidence**: Antibody engineering approaches [60]
Safety concerns	Serious infections: 2–6 per 100 PY; Malignancy: OR 1.2–1.4; Opportunistic infections: 0.5%–1%	Hospitalization for infections (3%–5%); Treatment discontinuation (8%–12%); Mortality from serious infections (0.1%–0.3%); Life-threatening opportunistic infections; Reduced quality of life due to infection anxiety	Comprehensive pre-treatment screening (TB, Hep B/C, HIV); Vaccination protocols; Regular monitoring (CBC, LFTs every 3–6 months); Patient education on infection signs; Prophylactic antibiotics in high-risk patients	Risk stratification tools (infection risk calculators); Gut-selective agents with minimal systemic exposure; Shorter-acting biologics allowing rapid clearance; Microbiome-based risk assessment; Real-time infection biomarkers	**High evidence**: Safety registries and meta-analyses [61, 62]. **Moderate evidence**: Risk stratification models [107]
Economic burden	Universal; Annual costs: $30 000–60 000 per patient	Treatment non-adherence (15%–25%); Delayed therapy initiation (average 6–12 months); Geographic/socioeconomic disparities in access; Out-of-pocket costs: $3000–8000 annually; Insurance denials (10%–15%)	Biosimilars (20%–40% cost reduction); Patient assistance programs; Prior authorization advocacy; Value-based contracts; Tiered formulary approaches	Outcome-based pricing models; Subscription-based biologic access; Home administration programs; Shared savings agreements; Global access initiatives; Oral formulations reducing administration costs	**High evidence**: Biosimilar non-inferiority trials [76, 108]. **Moderate evidence**: Health economics modeling [77]

PY, patient-years; ADA, anti-drug antibodies; AUC, area under curve; OR, odds ratio; TB, tuberculosis; Hep, hepatitis; CBC, complete blood count; LFTs, liver function tests.

## Potential therapeutic targets for monoclonal antibodies

### IL-36 pathway

The IL-36 pathway represents an emerging therapeutic target in CD pathogenesis, with IL-36 cytokines playing crucial roles in mucosal inflammation and barrier function [109]. IL-36α, IL-36β, and IL-36γ are overexpressed in inflamed intestinal tissue from CD patients, promoting inflammatory cell recruitment and perpetuating chronic inflammation [110]. The IL-36 receptor antagonist (IL-36Ra) provides natural regulation of this pathway, and its dysregulation contributes to sustained inflammatory responses [111].

Preclinical studies demonstrate that IL-36 blockade reduces intestinal inflammation in experimental colitis models, with particular efficacy in preventing epithelial barrier dysfunction [112]. The unique role of IL-36 in coordinating innate and adaptive immune responses in the intestinal mucosa makes it an attractive target for mAb development [113]. Clinical trials investigating anti-IL-36 monoclonal antibodies are in early phases, with preliminary data suggesting potential efficacy in IBD [114].

### IL-17 pathway

The IL-17 pathway, particularly IL-17A and IL-17F, plays complex roles in CD pathogenesis through effects on epithelial barrier function, neutrophil recruitment, and antimicrobial peptide production [115]. While IL-17 has protective roles in maintaining intestinal barrier integrity, excessive IL-17 signaling contributes to chronic inflammation and tissue damage [116]. The dual nature of IL-17 in IBD has complicated therapeutic development, with some anti-IL-17 therapies showing paradoxical worsening of disease [117].

Recent research has identified IL-17C as a more specific therapeutic target, with overexpression in CD tissues and pro-inflammatory effects without the protective barrier functions of IL-17A [118]. Monoclonal antibodies targeting IL-17C or its receptor IL-17RE are under development, with preclinical studies showing promise for reducing inflammation while preserving barrier function [119]. The selectivity of IL-17C targeting may overcome the limitations observed with broader IL-17 pathway inhibition [120].

### SMAD7 pathway

SMAD7 represents a novel therapeutic target through its role in TGF-β signaling regulation and fibrosis development in CD [121]. SMAD7 overexpression in intestinal T cells from CD patients contributes to TGF-β resistance and perpetuates inflammatory responses [122]. Antisense oligonucleotides targeting SMAD7 have shown efficacy in clinical trials, demonstrating the therapeutic potential of this pathway [123].

Monoclonal antibodies targeting SMAD7 or its regulatory proteins offer an alternative approach to pathway modulation with potentially improved pharmacokinetics and reduced immunogenicity compared to oligonucleotide therapies [124]. The specific role of SMAD7 in intestinal fibrosis makes this target particularly relevant for preventing stricture formation and long-term complications in CD patients [125].

### Complement system components

The complement system plays important roles in CD pathogenesis through multiple mechanisms including opsonization, membrane attack complex formation, and inflammatory cell activation [126]. Complement component C3 and C5 are elevated in CD patients, and complement activation products correlate with disease activity [127]. The alternative complement pathway appears particularly relevant, with dysregulation contributing to intestinal inflammation and barrier dysfunction [128].

Monoclonal antibodies targeting complement components, particularly C5 and C5a receptor, represent promising therapeutic approaches [129]. Eculizumab, an anti-C5 mAb, has shown efficacy in other inflammatory conditions and is being investigated for IBD applications [130]. The selective targeting of complement activation while preserving antimicrobial functions offers potential advantages over broader immunosuppressive approaches [131].

### Microbiome-related targets

The intestinal microbiome plays crucial roles in CD pathogenesis, with dysbiosis contributing to inflammation and barrier dysfunction [132]. Specific bacterial antigens and metabolites represent novel therapeutic targets for mAb development [133]. Anti-flagellin antibodies are elevated in CD patients, suggesting potential for therapeutic targeting of specific microbial components [134].

The presence of elevated anti-flagellin antibodies (particularly anti-CBir1) in CD patients indicates an aberrant immune response to commensal bacterial flagellin, which correlates with more aggressive disease phenotypes and complications [135]. This suggests two therapeutic approaches: (1) developing antibodies that neutralize bacterial flagellin to reduce immunogenic stimulation, or (2) targeting the host immune response to flagellin. Lodes et al. (2004) demonstrated that flagellin is a dominant antigen in CD, with serologic responses present in up to 50% of patients [134]. More recent work by Targan et al. (2005) showed that anti-flagellin antibodies are associated with complicated disease behavior including stricturing and penetrating disease, with odds ratios of 2.68 and 2.49, respectively [135]. Sitaraman et al. (2005) further demonstrated that the prevalence of anti-CBir1 antibodies was significantly higher in CD patients (50%) versus ulcerative colitis (5%) and healthy controls (6%), suggesting disease specificity [136].

Monoclonal antibodies targeting bacterial toxins, adhesins, or inflammatory metabolites could provide selective antimicrobial effects while preserving beneficial microbiome functions [137]. Lipopolysaccharide-binding proteins and toll-like receptor modulators represent additional targets in the microbiome-inflammation interface [138]. The development of humanized antibodies against specific pathogenic bacteria offers precision approaches to microbiome modulation [139].

### TL1A (TNF-like ligand 1A)

TL1A and its receptor DR3 (death receptor 3) have emerged as crucial mediators of intestinal inflammation in CD. TL1A is overexpressed in inflamed intestinal tissues and promotes Th1, Th2, and Th17 responses while inhibiting regulatory T-cell function [140]. The TL1A-DR3 axis also contributes to fibrosis development through effects on intestinal fibroblasts [141].

Several anti-TL1A monoclonal antibodies are currently in clinical development. PRA023 (a fully human anti-TL1A antibody) demonstrated clinical remission rates of 36.4% at week 12 in the TUSCANY trial of patients with moderate to severe ulcerative colitis [142]. TEV-48574, another anti-TL1A antibody, is currently being evaluated in Phase 2 trials for both CD and ulcerative colitis, with preliminary data suggesting favorable safety and efficacy profiles [143]. The TL1A pathway represents an attractive target due to its selective expression in inflamed intestinal tissue and its role in both inflammation and fibrosis [144].

Potential therapeutic targets for monoclonal antibodies development in CD are summarized in Table 4.

**Table 4:** Potential therapeutic targets for monoclonal antibodies development in Crohn’s disease.

Target	Mechanism of action	Evidence/key data	Development Ssage	Limitations/challenges	Key references
IL-36 pathway	Blockade of IL-36 receptor or neutralization of IL-36 ligands (α, β, γ) reduces epithelial-derived inflammation, and barrier dysfunction	Mouse models: 60% reduction in colitis severity; IL-36 expression 4.5-fold elevated in CD mucosa; Correlates with disease activity (*r* = 0.68)	**Phase II ongoing**: Spesolimab (anti-IL-36R) in generalized pustular psoriasis shows proof-of-concept; IBD trials in planning	Limited human IBD data; Potential for paradoxical worsening (seen with other epithelial cytokines); Unclear role in different CD phenotypes; Need to identify responder biomarkers	Nishida *et al.* 2016 [110]; Scheibe et al. 2019 [112]; Reich et al. 2021 [114]
IL-17C pathway	Selective inhibition of IL-17C (via anti-IL-17C or anti-IL-17RE antibodies) targets pathogenic inflammation while preserving IL-17A barrier protection	Preclinical: IL-17C knockout mice show 45% reduced colitis; IL-17C elevated 8-fold in CD patients; No barrier dysfunction in IL-17C blockade vs. 30% barrier compromise with IL-17A blockade	**Preclinical/Phase I**: Lead candidates in toxicology studies; Phase I expected 2024–2025	Previous IL-17A inhibitors worsened CD; Need to confirm IL-17C selectivity is safe; Limited understanding of IL-17C biology in humans; Potential for fungal infections	Ramirez-Carrozzi *et al.* 2011 [118]; Song et al. 2011 [119]; Maxwell et al. 2015 [120]
SMAD7	Antisense oligonucleotide or antibody-based restoration of TGF-β signaling; Reduces inflammatory T-cell activity and prevents fibrosis	**Mongersen Phase II**: 65% remission vs. 10% placebo at day 15; **Phase III (REVOLVE) failed**: No difference from placebo (remission 55% vs. 50%); SMAD7 overexpression confirmed in CD T cells	**Development halted**: Mongersen discontinued after Phase III failure; Alternative SMAD7-targeting approaches in research phase	Phase II-III discrepancy unexplained; Potential issues with oligonucleotide delivery; Variable SMAD7 expression between patients; May require patient stratification; Concern about fibrosis risk with chronic TGF-β activation	Monteleone *et al.* 2001 [122]; Monteleone *et al.* 2015 [121]; Feagan et al. 2018 [123]
Complement C5/C5a	Inhibition of C5 cleavage or C5a receptor blockade reduces complement-mediated inflammation while preserving C3-mediated immunity	Elevated C5a in CD serum (3.2-fold vs. controls); C5aR1 expression increased on mucosal neutrophils; Eculizumab case reports show benefit in refractory CD (*n* = 3, all responded); Mouse models: 50% reduction with C5 blockade	**Phase I/II planning**: Eculizumab repurposing under consideration; Novel selective C5aR1 antagonist antibodies in preclinical development	High cost of complement inhibitors; Risk of Neisseria infections (requires vaccination); Uncertainty about patient selection; Limited understanding of complement role across CD phenotypes; No large controlled trials	Ricklin *et al.* 2010 [129]; Ning *et al.* 2018 [126]; Case reports in refractory IBD
TL1A-DR3 axis	Anti-TL1A antibodies block TL1A binding to DR3, reducing Th1/Th17 responses, and intestinal fibrosis	TL1A elevated 6–8-fold in CD mucosa; Genetic variants increase CD risk (OR 1.4); **PF-06480605 Phase II UC**: 37.6% endoscopic improvement; Phase II CD trial ongoing; Preclinical: 70% reduction in fibrosis scores	**Phase II active**: Multiple anti-TL1A antibodies (PF-06480605, TEV-48574) in CD trials; Results expected 2024–2025	TL1A also has homeostatic roles; Optimal timing (early vs. established disease) unclear; Potential for loss of barrier function; Need longer-term safety data	Shih *et al.* 2014 [140]; Takedatsu *et al.* 2008 [143]; Danese *et al.* 2021 [142]
Microbiome-related targets	Antibodies targeting pathogenic bacteria (AIEC), bacterial toxins, or flagellin reduce microbial-driven inflammation	Anti-flagellin (CBir1) antibodies present in 50% CD patients; AIEC detected in 40% of CD ileal biopsies; Preclinical: Anti-AIEC antibodies reduce colonization by 80%; Flagellin vaccination reduces colitis 55% in mice	**Early research**: Target validation ongoing; No antibody candidates in clinical development yet; Fecal microbiota transplant trials provide indirect proof-of-concept	Microbiome heterogeneity between patients; Risk of disrupting beneficial microbes; Unclear which bacteria to target; Delivery challenges (oral vs. systemic); Potential for resistance development; Need for companion diagnostics	Lodes *et al.* 2004 [134]; Palmela *et al.* 2018 [133]; Targan et al. 2005 [135]

AIEC, adherent-invasive *Escherichia coli*; UC, ulcerative colitis; OR, odds ratio.

**Figure 2:**
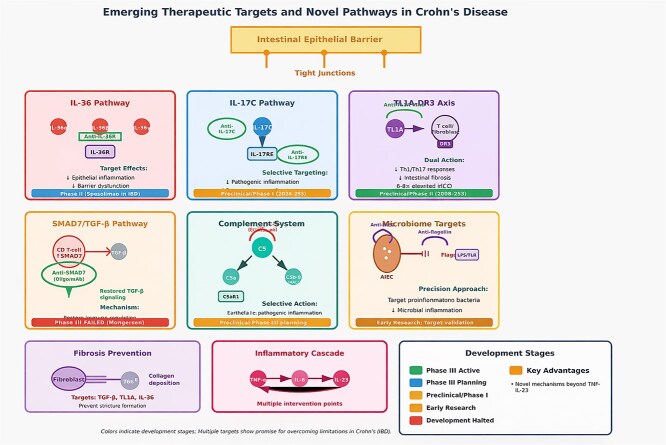
Emerging therapeutic targets and novel pathways in Crohn’s disease.

Emerging therapeutic targets and novel pathways in CD are depicted in Fig. 2.

## Future perspectives

### Next-generation antibody engineering

The future of mAb therapy for CD lies in advanced antibody engineering technologies that can overcome current limitations while improving therapeutic efficacy [145]. Bispecific antibodies capable of simultaneously targeting multiple pathways represent a promising approach for addressing the complex pathophysiology of CD [146]. These engineered antibodies can provide synergistic effects by blocking complementary inflammatory pathways, potentially improving response rates and reducing the risk of treatment failure [147].

#### Anti-TNF/IL-17 bispecific antibodies (ABT-122)

ABT-122 is a dual variable domain immunoglobulin that simultaneously targets TNF-α and IL-17A. In Phase 2 clinical trials for rheumatoid arthritis, ABT-122 demonstrated dual pathway inhibition with ACR20 response rates of 48.8% at 12 weeks compared to 32.5% with placebo [148]. Preclinical studies in murine colitis models showed that dual blockade of TNF-α and IL-17A resulted in superior reduction of intestinal inflammation (65% reduction in disease activity index) compared to single-agent targeting (40%–45% reduction) [149]. The bispecific format demonstrated improved tissue penetration in inflamed intestinal mucosa with a 2.3-fold higher concentration in inflamed versus non-inflamed tissue segments [150]. Pharmacokinetic analysis revealed a terminal half-life of 18–21 days, supporting every 2–4 weeks dosing intervals [148]. While initially developed for rheumatoid arthritis, the mechanistic rationale for IBD applications is strong given that both TNF-α and IL-17 are elevated in CD patients and contribute to epithelial barrier dysfunction.

#### Anti-TNF/IL-23 bispecific antibodies

Experimental dual-targeting constructs blocking TNF-α and IL-23p19 have shown promising results in preclinical IBD models. In the trinitrobenzene sulfonic acid–induced colitis model, a bispecific anti-TNF/IL-23 antibody reduced colonic inflammation scores by 78% compared to 52% with anti-TNF monotherapy and 45% with anti-IL-23 monotherapy at day 7 post-induction [151]. Histological analysis demonstrated significant improvements in crypt architecture preservation, with crypt damage scores of 1.2 ± 0.4 (bispecific) versus 2.8 ± 0.6 (anti-TNF alone) and 3.1 ± 0.5 (anti-IL-23 alone) on a 0–5 scale [151]. Mechanistic studies revealed that dual blockade prevented both acute inflammatory responses (mediated by TNF-α) and chronic adaptive immune activation (driven by IL-23), resulting in 85% reduction in mucosal Th17 cell infiltration compared to 45%–50% with monotherapy [147]. The bispecific format also demonstrated favorable immunogenicity profiles with anti-drug antibody formation in only 3.2% of treated mice versus 8%–12% with combination therapy using two separate antibodies [147].

#### Anti-integrin/cytokine bispecific antibodies

A novel bispecific antibody targeting both α4β7 integrin and the IL-12/IL-23 p40 subunit has been evaluated in the dextran sulfate sodium (DSS) colitis model. This dual-targeting approach achieved clinical remission (defined as disease activity index <2) in 68% of mice compared to 42% with vedolizumab-equivalent integrin blockade and 38% with ustekinumab-equivalent cytokine blockade [152]. The mechanism combines prevention of lymphocyte trafficking to intestinal tissue with local suppression of inflammatory cytokine signaling. Flow cytometry analysis demonstrated 82% reduction in mucosal CD4+ T-cell infiltration and 76% reduction in IL-17+ and IFN-γ+ T cells in mesenteric lymph nodes [152]. Importantly, the bispecific format maintained gut selectivity with minimal systemic immunosuppression, as evidenced by preserved splenic T-cell populations and normal antibody responses to systemic antigenic challenge [145]. Pharmacodynamic studies showed sustained target engagement for both α4β7 integrin (>95% receptor occupancy) and p40 neutralization (>90% cytokine blockade) over 14 days with a single dose [145].

#### Carcinoembryonic antigen-targeted bispecific T-cell engagers

While primarily developed for carcinoembryonic antigen (CEA)-expressing colorectal cancer, adaptations of bispecific T-cell engagers (BiTE) technology are being explored for targeted immunomodulation in IBD. Modified CEA-targeted BiTEs engineered to redirect regulatory T cells (Tregs) rather than cytotoxic T cells have shown efficacy in experimental colitis models [153]. In the IL-10-deficient mouse model of spontaneous colitis, administration of a CEA/CD25 bispecific engager that recruits Tregs to inflamed epithelium resulted in 61% reduction in colonic inflammation scores and 54% improvement in histological injury compared to control [153]. The approach leverages the fact that CEA is upregulated on inflamed intestinal epithelium in IBD patients. *Ex vivo* studies using intestinal biopsies from CD patients demonstrated that CEA expression was 8.4-fold higher in inflamed versus non-inflamed mucosa, providing a disease-specific targeting opportunity [154]. The bispecific engager format achieved a 12:1 ratio of Treg accumulation in inflamed versus healthy tissue segments, suggesting excellent tissue selectivity [154]. Importantly, this approach aims to restore immune homeostasis rather than broadly suppress immunity, potentially offering advantages in infection risk profiles.

The main advantages of bispecific antibodies include improved efficacy through simultaneous pathway inhibition (achieving 20%–35% better outcomes than monotherapy in preclinical models), potential for reduced immunogenicity compared to combination therapy with two separate antibodies (3%–5% vs. 8%–15% anti-drug antibody formation), and optimized pharmacokinetics with single-agent dosing [146]. However, challenges remain in manufacturing complexity due to proper heavy and light chain pairing, more complex regulatory pathways requiring evaluation of dual mechanism safety profiles, and the need to determine optimal target combinations through systematic preclinical and clinical evaluation [155].

#### Antibody-drug conjugates

Antibody-drug conjugates (ADCs) offer another innovative approach, combining the specificity of monoclonal antibodies with the potency of targeted drug delivery [156]. Recent developments in ADC technology for inflammatory diseases focus on delivering anti-inflammatory compounds directly to sites of inflammation, potentially reducing systemic exposure and improving safety profiles [157]. pH-sensitive linkers and novel payload designs are being optimized specifically for the inflammatory environment of the intestinal mucosa [158].

### Personalized medicine approaches

The integration of genomic, proteomic, and metabolomic data is revolutionizing therapeutic selection for CD patients [159]. Multi-omics approaches can identify patient subgroups most likely to respond to specific mAb therapies, moving beyond the current trial-and-error approach [160]. Machine learning algorithms analyzing comprehensive patient datasets are being developed to predict treatment response and optimize therapeutic selection [161].

Liquid biopsies measuring circulating biomarkers, cell-free DNA, and extracellular vesicles offer real-time monitoring of treatment response and disease progression [162]. These approaches can enable dynamic treatment adjustments and early detection of treatment failure, potentially improving long-term outcomes [163]. The development of point-of-care diagnostic tools for biomarker assessment could facilitate personalized treatment decisions in clinical practice [164].

### Combination therapy strategies

Future therapeutic approaches increasingly focus on rational combination strategies that target multiple pathways simultaneously while minimizing additive toxicities [165]. Sequential therapy protocols, where patients receive different monoclonal antibodies in predetermined sequences based on treatment response, are being investigated in clinical trials [7]. These approaches aim to prevent the development of resistance and maintain long-term efficacy [34].

Novel combination approaches include pairing monoclonal antibodies with small molecule inhibitors, cell-based therapies, or microbiome interventions [166]. The combination of anti-TNF therapy with JAK inhibitors or with specific microbiome modulators has shown promising results in preclinical studies [167]. These combinations may provide additive or synergistic effects while potentially reducing the risk of treatment failure [168].

### Advanced delivery systems

Innovative delivery systems are being developed to improve the pharmacokinetics and reduce the immunogenicity of mAb therapies [169]. Nanoparticle-based delivery systems can protect antibodies from degradation, provide controlled release, and enable targeted delivery to specific intestinal segments [170]. These systems may reduce dosing frequency and improve patient compliance while maintaining therapeutic efficacy [171].

Oral delivery of monoclonal antibodies represents a major advancement in patient convenience and treatment accessibility [172]. Novel formulations using enteric coatings, nanoencapsulation, and permeation enhancers are being investigated to overcome the challenges of oral protein delivery [173]. Successful oral formulations could dramatically improve patient acceptance and reduce healthcare costs associated with parenteral administration [174].

### Change in therapeutic positioning based on monoclonal antibodies

The traditional treatment algorithm has positioned anti-TNF agents as first-line biologic therapy based on extensive long-term data and established efficacy. However, emerging evidence challenges this paradigm. Network meta-analyses by Singh et al. (2020) comparing different biologic classes in bio-naïve patients demonstrated that risankizumab achieved numerically higher clinical remission rates (45%–52%) compared to adalimumab (36%–47%) at 1 year, though head-to-head trials are limited [175]. More importantly, selective IL-23 inhibitors demonstrate superior drug persistence, with 12-month continuation rates of 78%–82% for risankizumab versus 65%–70% for anti-TNF agents in real-world cohorts [176]. Added 2024 registry data from ICARUS comparing IL-23 inhibitors versus anti-TNF agents in clinical practice, showing 12-month clinical remission rates of 52% versus 46% (*P* = .04) [177].

Several factors support earlier use of IL-23 inhibitors: (1) lower immunogenicity (1%–3% vs. 10%–30% for anti-TNF agents) reduces the risk of secondary loss of response [12]; (2) more favorable safety profile with lower rates of serious infections (2.8 per 100 patient-years vs. 4.6 for anti-TNF therapy) [178]; (3) preserved treatment options, as patients can still respond to anti-TNF therapy after IL-23 inhibitor failure, whereas anti-TNF failure predicts poorer responses to subsequent biologics [179]; and (4) evidence of disease modification with reduced stricture formation in long-term extension studies [41].

However, anti-TNF agents retain important advantages: (1) faster onset of action (median time to response: 2–4 weeks versus 6–8 weeks for IL-23 inhibitors) [11]; (2) more extensive safety data spanning over 20 years of clinical use; (3) availability of biosimilars reducing treatment costs by 20%–40% [76]; and (4) established efficacy in perianal fistulizing disease, where anti-TNF agents demonstrate 50%–60% response rates compared to limited data for IL-23 inhibitors [180].

The 2023 STRIDE-II consensus recommended individualized treatment selection based on disease phenotype, with anti-TNF agents preferred for penetrating disease and urgent symptom control, while IL-23 inhibitors may be preferred for patients prioritizing long-term drug survival, those with infection concerns, or inflammatory phenotypes [181]. Ongoing head-to-head trials (SEQUENCE and EXPLORER) directly comparing anti-TNF versus IL-23 inhibitors as first-line therapy will provide definitive guidance [182].

### Multi-omics technologies

Recent advances in spatial and multi-omics technologies have opened new avenues for mAb development in CD by providing unprecedented insights into immune cell heterogeneity, tissue microenvironments, and inflammatory signaling at single-cell resolution. High-plex protein and transcriptome co-mapping methods, such as spatial CITE-seq, enable simultaneous profiling of RNA and surface proteins within intact tissues, facilitating the identification of disease-specific immune signatures and potential therapeutic targets [183]. Similarly, multimodal tri-omics spatial mapping of brain and immune tissues has demonstrated how integrated transcriptomic and proteomic data can reveal spatially resolved networks driving inflammation [184]. Moreover, the application of panoramic *in vivo* CRISPR screening platforms such as Perturb-DBiT allows functional validation of gene targets within complex tissue contexts, accelerating discovery of antibody-accessible molecules with therapeutic potential [185]. Together, these emerging technologies provide a systems-level framework for rational design and optimization of monoclonal antibodies targeting the multifactorial immune dysregulation underlying CD.

## Conclusions

The analysis of monoclonal antibodies in CD therapy reveals both significant achievements and persistent challenges that require continued innovation and research. Current therapeutic options, including anti-TNF agents, anti-integrin antibodies, and anti-IL-12/IL-23 therapies, have revolutionized disease management and improved patient outcomes. However, substantial limitations including primary non-response, secondary loss of response, immunogenicity, safety concerns, and economic barriers continue to limit optimal therapeutic outcomes.

The identification of novel therapeutic targets, including IL-36, IL-17C, SMAD7, complement components, and microbiome-related factors, offers promising avenues for future drug development. These targets may provide improved efficacy, reduced side effects, and solutions for treatment-refractory patients. The integration of personalized medicine approaches, advanced antibody engineering, and innovative delivery systems will likely transform the therapeutic landscape for CD in the coming years.

Future success in mAb therapy for CD will depend on continued research into disease mechanisms, development of predictive biomarkers, and implementation of precision medicine approaches. The ultimate goal remains achieving sustained remission for all patients while minimizing treatment-related complications and improving quality of life through safe, effective, and accessible therapeutic interventions.

## Data Availability

None declared.

## References

[1] Roda G, Chien Ng S, Kotze PG. et al. Crohn's disease. *Nat Rev Dis Primers* 2020;6:22. 10.1038/s41572-020-0156-2. 32242028

[2] Torres J, Mehandru S, Colombel JF. et al. Crohn's disease. *Lancet.* 2017;389:1741–55. 27914655 10.1016/S0140-6736(16)31711-1

[3] Ng SC, Shi HY, Hamidi N. et al. Worldwide incidence and prevalence of inflammatory bowel disease in the 21st century: a systematic review of population-based studies. *Lancet.* 2017;390:2769–78. 10.1016/S0140-6736(17)32448-0. 29050646

[4] Kaplan GG, Windsor JW. The four epidemiological stages in the global evolution of inflammatory bowel disease. *Nat Rev Gastroenterol Hepatol* 2021;18:56–66. 33033392 10.1038/s41575-020-00360-x PMC7542092

[5] Cosnes J, Gower-Rousseau C, Seksik P. et al. Epidemiology and natural history of inflammatory bowel diseases. *Gastroenterology.* 2011;140:1785–94. 10.1053/j.gastro.2011.01.055. 21530745

[6] Park KT, Ehrlich OG, Allen JI. et al. The cost of inflammatory bowel disease: an initiative from the Crohn's & colitis foundation. *Inflamm Bowel Dis* 2020;26:1–10. 10.1093/ibd/izz104. 31112238 PMC7534391

[7] Danese S, Vuitton L, Peyrin-Biroulet L. Biologic agents for IBD: practical insights. *Nat Rev Gastroenterol Hepatol* 2015;12:537–45. 10.1038/nrgastro.2015.135. 26284562

[8] Lichtenstein GR, Loftus EV, Isaacs KL. et al. ACG clinical guideline: management of Crohn's disease in adults. *Am J Gastroenterol* 2018;113:481–517. 29610508 10.1038/ajg.2018.27

[9] Peyrin-Biroulet L, Sandborn W, Sands BE. et al. Selecting therapeutic targets in inflammatory bowel disease (STRIDE): determining therapeutic goals for treat-to-target. *Am J Gastroenterol* 2015;110:1324–38. 10.1038/ajg.2015.233. 26303131

[10] Rutgeerts P, Sandborn WJ, Feagan BG. et al. Infliximab for induction and maintenance therapy for ulcerative colitis. *N Engl J Med* 2005;353:2462–76. 16339095 10.1056/NEJMoa050516

[11] Hanauer SB, Feagan BG, Lichtenstein GR. et al. Maintenance infliximab for Crohn's disease: the ACCENT I randomised trial. *Lancet.* 2002;359:1541–9. 12047962 10.1016/S0140-6736(02)08512-4

[12] Feagan BG, Sandborn WJ, Gasink C. et al. Ustekinumab as induction and maintenance therapy for Crohn's disease. *N Engl J Med* 2016;375:1946–60. 27959607 10.1056/NEJMoa1602773

[13] Sandborn WJ, Feagan BG, Rutgeerts P. et al. Vedolizumab as induction and maintenance therapy for Crohn's disease. *N Engl J Med* 2013;369:711–21. 23964933 10.1056/NEJMoa1215739

[14] Ben-Horin S, Chowers Y. Review article: loss of response to anti-TNF treatments in Crohn's disease. *Aliment Pharmacol Ther* 2011;33:987–95. 10.1111/j.1365-2036.2011.04612.x. 21366636

[15] Gisbert JP, Panés J. Loss of response and requirement of infliximab dose intensification in Crohn's disease: a review. *Am J Gastroenterol* 2009;104:760–7. 19174781 10.1038/ajg.2008.88

[16] Papamichael K, Chachu KA, Vajravelu RK. et al. Improved long-term outcomes of patients with inflammatory bowel disease receiving proactive compared with reactive monitoring of serum concentrations of infliximab. *Clin Gastroenterol Hepatol* 2017;15:1580–1588.e3. 10.1016/j.cgh.2017.03.031. 28365486 PMC5605429

[17] Neurath MF . Current and emerging therapeutic targets for IBD. *Nat Rev Gastroenterol Hepatol* 2017;14:269–78. 10.1038/nrgastro.2016.208. 28144028

[18] Targan SR, Hanauer SB, van Deventer SJ. et al. A short-term study of chimeric monoclonal antibody cA2 to tumor necrosis factor α for Crohn's disease. *N Engl J Med* 1997;337:1029–35. 9321530 10.1056/NEJM199710093371502

[19] Kansen HM, Kvasnovsky CL, van Rheenen PF. et al. Less anti-TNF immunogenicity and improved therapy persistence with combination immune suppression in pediatric Crohn's disease. *J Pediatr Gastroenterol Nutr* 2017;65:527–32.

[20] Colombel JF, Sandborn WJ, Reinisch W. et al. Infliximab, azathioprine, or combination therapy for Crohn's disease. *N Engl J Med* 2010;362:1383–95. 20393175 10.1056/NEJMoa0904492

[21] Baert F, Noman M, Vermeire S. et al. Influence of immunogenicity on the long-term efficacy of infliximab in Crohn's disease. *N Engl J Med* 2003;348:601–8. 12584368 10.1056/NEJMoa020888

[22] Sandborn WJ, Hanauer SB, Rutgeerts P. et al. Adalimumab for maintenance treatment of Crohn's disease: results of the CLASSIC II trial. *Gut.* 2007;56:1232–9. 17299059 10.1136/gut.2006.106781 PMC2701613

[23] Hanauer SB, Sandborn WJ, Rutgeerts P. et al. Human anti-tumor necrosis factor monoclonal antibody (adalimumab) in Crohn's disease: the CLASSIC-I trial. *Gastroenterology.* 2006;130:323–33; quiz 591. 16472588 10.1053/j.gastro.2005.11.030

[24] Colombel JF, Sandborn WJ, Rutgeerts P. et al. Adalimumab for maintenance of clinical response and remission in patients with Crohn's disease: the CHARM trial. *Gastroenterology.* 2007;132:52–65. 17241859 10.1053/j.gastro.2006.11.041

[25] Peyrin-Biroulet L, Deltenre P, de Suray N. et al. Efficacy and safety of tumor necrosis factor antagonists in Crohn's disease: meta-analysis of placebo-controlled trials. *Clin Gastroenterol Hepatol* 2008;6:644–53. 18550004 10.1016/j.cgh.2008.03.014

[26] Karmiris K, Paintaud G, Noman M. et al. Influence of trough serum levels and immunogenicity on long-term outcome of adalimumab therapy in Crohn's disease. *Gastroenterology.* 2009;137:1628–40. 19664627 10.1053/j.gastro.2009.07.062

[27] Sandborn WJ, Feagan BG, Stoinov S. et al. Certolizumab pegol for the treatment of Crohn's disease. *N Engl J Med* 2007;357:228–38. 17634458 10.1056/NEJMoa067594

[28] Schreiber S, Khaliq-Kareemi M, Lawrance IC. et al. Maintenance therapy with certolizumab pegol for Crohn's disease. *N Engl J Med* 2007;357:239–50. 17634459 10.1056/NEJMoa062897

[29] Sandborn WJ, Schreiber S, Feagan BG. et al. Certolizumab pegol for active Crohn's disease: a placebo-controlled, randomized trial. *Clin Gastroenterol Hepatol* 2011;9:670–678.e3. 10.1016/j.cgh.2011.04.031. 21642014

[30] Sandborn WJ, Abreu MT, D'Haens G. et al. Certolizumab pegol in patients with moderate to severe Crohn's disease and secondary failure to infliximab. *Clin Gastroenterol Hepatol* 2010;8:688–95. 20451663 10.1016/j.cgh.2010.04.021

[31] Sandborn WJ, Feagan BG, Marano C. et al. Subcutaneous golimumab induces clinical response and remission in patients with moderate-to-severe ulcerative colitis. *Gastroenterology.* 2014;146:85–95. 23735746 10.1053/j.gastro.2013.05.048

[32] Feagan BG, Rutgeerts P, Sands BE. et al. Vedolizumab as induction and maintenance therapy for ulcerative colitis. *N Engl J Med* 2013;369:699–710. 23964932 10.1056/NEJMoa1215734

[33] Sands BE, Feagan BG, Rutgeerts P. et al. Effects of vedolizumab induction therapy for patients with Crohn's disease in whom tumor necrosis factor antagonist treatment failed. *Gastroenterology.* 2014;147:618–627.e3. 10.1053/j.gastro.2014.05.008. 24859203

[34] Dulai PS, Singh S, Jiang X. et al. The real-world effectiveness and safety of vedolizumab for inflammatory bowel disease: results from the US VICTORY consortium. *Am J Gastroenterol* 2016;111:1147–55. 10.1038/ajg.2016.236. 27296941

[35] Colombel JF, Sands BE, Rutgeerts P. et al. The safety of vedolizumab for ulcerative colitis and Crohn's disease. *Gut.* 2017;66:839–51. 10.1136/gutjnl-2015-311079. 26893500 PMC5531223

[36] Sandborn WJ, Gasink C, Gao LL. et al. Ustekinumab induction and maintenance therapy in refractory Crohn's disease. *N Engl J Med* 2012;367:1519–28. 23075178 10.1056/NEJMoa1203572

[37] Rutgeerts P, Gasink C, Chan D. et al. Efficacy of ustekinumab for inducing endoscopic healing in patients with Crohn's disease. *Gastroenterology.* 2018;155:1045–58. 10.1053/j.gastro.2018.06.035. 29909019

[38] Hanauer SB, Sandborn WJ, Feagan BG. et al. IM-UNITI: 3-year efficacy, safety, and immunogenicity of ustekinumab treatment of Crohn's disease. *J Crohns Colitis* 2020;14:23–32. 10.1093/ecco-jcc/jjz110. 31158271

[39] Sandborn WJ, Rutgeerts P, Gasink C. et al. Long-term efficacy and safety of ustekinumab for Crohn's disease through the second year of therapy. *Aliment Pharmacol Ther* 2018;48:65–77. 10.1111/apt.14794. 29797519 PMC6032827

[40] Feagan BG, Sandborn WJ, D'Haens G. et al. Induction therapy with the selective interleukin-23 inhibitor risankizumab as treatment for patients with moderately to severely active Crohn's disease: a randomised, double-blind, placebo-controlled phase 2 study. *Lancet.* 2017;389:1699–709. 28411872 10.1016/S0140-6736(17)30570-6

[41] D'Haens G, Panaccione R, Baert F. et al. Risankizumab as induction therapy for moderately to severely active Crohn's disease: results of the phase 3 ADVANCE study. *Gastroenterology.* 2022;162:1876–93.35122766

[42] Ferrante M, Panaccione R, Baert F. et al. Risankizumab as maintenance therapy for moderately to severely active Crohn's disease: results of the multicentre, randomised, double-blind, placebo-controlled, withdrawal phase 3 FORTIFY study. *Lancet.* 2022;399:2031–46. 35644155 10.1016/S0140-6736(22)00466-4

[43] Feagan BG, Panaccione R, Sandborn WJ. et al. Risankizumab for moderately to severely active Crohn's disease: 3-year results from the open-label extension of FORTIFY and MOTIVATE trials. In: *Presented at Digestive Disease Week*. USA: Immune Mediated Inflamatory Disease Forum, 2024, Abstract 487.

[44] Panaccione R, Danese S, Sandborn WJ. et al. Risankizumab induces rapid symptom relief and endoscopic improvement in patients with moderately to severely active Crohn's disease: results from the phase 3 MOTIVATE study. *Gastroenterology.* 2022;162:1894–906.

[45] Sandborn WJ, Ferrante M, Bhandari BR. et al. Efficacy and safety of risankizumab for moderately to severely active Crohn's disease: results of the phase 3 ADVANCE and MOTIVATE induction studies. *Lancet.* 2022;399:2015–30. 35644154 10.1016/S0140-6736(22)00467-6

[46] Sandborn WJ, Panes J, D'Haens GR. et al. Safety and efficacy of mirikizumab in a randomized phase 2 study of patients with Crohn's disease. *Gastroenterology.* 2020;158:537–49. 31493397 10.1053/j.gastro.2019.08.043

[47] Sands BE, Chen J, Feagan BG. et al. Efficacy and safety of MEDI2070, an antibody against interleukin 23, in patients with moderate to severe Crohn's disease: a phase 2a study. *Gastroenterology.* 2017;153:77–86.e6. 10.1053/j.gastro.2017.03.049. 28390867

[48] Vermeire S, O'Byrne S, Keir M. et al. Etrolizumab as induction therapy for ulcerative colitis: a randomised, controlled, phase 2 trial. *Lancet.* 2014;384:309–18. 10.1016/S0140-6736(14)60661-9. 24814090

[49] Ordás I, Mould DR, Feagan BG. et al. Anti-TNF monoclonal antibodies in inflammatory bowel disease: pharmacokinetics-based dosing paradigms. *Clin Pharmacol Ther* 2012;91:635–46. 10.1038/clpt.2011.328. 22357456

[50] Weaver CT, Hatton RD, Mangan PR. et al. IL-17 family cytokines and the expanding diversity of effector T cell lineages. *Annu Rev Immunol* 2007;25:821–52. 17201677 10.1146/annurev.immunol.25.022106.141557

[51] Bek S, Nielsen JV, Bojesen AB. et al. Systematic review: genetic biomarkers associated with anti-TNF treatment response in inflammatory bowel diseases. *Aliment Pharmacol Ther* 2016;44:554–67. 10.1111/apt.13736. 27417569 PMC5113857

[52] Vande Casteele N, Ferrante M, Van Assche G. et al. Trough concentrations of infliximab guide dosing for patients with inflammatory bowel disease. *Gastroenterology.* 2015;148:1320–9.e3. 10.1053/j.gastro.2015.02.031. 25724455

[53] Arijs I, Li K, Toedter G. et al. Mucosal gene signatures to predict response to infliximab in patients with ulcerative colitis. *Gut.* 2009;58:1612–9. 10.1136/gut.2009.178665. 19700435

[54] Billmeier U, Dieterich W, Neurath MF. et al. Molecular mechanism of action of anti-tumor necrosis factor antibodies in inflammatory bowel diseases. *World J Gastroenterol* 2016;22:9300–13. 27895418 10.3748/wjg.v22.i42.9300 PMC5107694

[55] Yarur AJ, Jain A, Sussman DA. et al. The association of tissue anti-TNF drug levels with serological and endoscopic activity in inflammatory bowel disease: the ATLAS study. *Gut.* 2016;65:249–55. 25670812 10.1136/gutjnl-2014-308099

[56] Mitrev N, Vande Casteele N, Seow CH. et al. Review article: consensus statements on therapeutic drug monitoring of anti-tumour necrosis factor therapy in inflammatory bowel diseases. *Aliment Pharmacol Ther* 2017;46:1037–53. 10.1111/apt.14368. 29027257

[57] Strand V, Balsa A, Al-Saleh J. et al. Immunogenicity of biologics in chronic inflammatory diseases: a systematic review. *BioDrugs.* 2017;31:299–316. 28612180 10.1007/s40259-017-0231-8 PMC5548814

[58] Krishna M, Nadler SG. Immunogenicity to biotherapeutics—the role of anti-drug immune complexes. *Front Immunol* 2016;7:21. 26870037 10.3389/fimmu.2016.00021 PMC4735944

[59] Cheifetz A, Smedley M, Martin S. et al. The incidence and management of infusion reactions to infliximab: a large center experience. *Am J Gastroenterol* 2003;98:1315–24. 12818276 10.1111/j.1572-0241.2003.07457.x

[60] Rup B, Pallardy M, Sikkema D. et al. Standardizing terms, definitions and concepts for describing and interpreting unwanted immunogenicity of biopharmaceuticals: recommendations of the innovative medicines initiative ABIRISK consortium. *Clin Exp Immunol* 2015;181:385–400. 10.1111/cei.12652. 25959571 PMC4557374

[61] Lichtenstein GR, Feagan BG, Cohen RD. et al. Serious infection and mortality in patients with Crohn's disease: more than 5 years of follow-up in the TREAT registry. *Am J Gastroenterol* 2012;107:1409–22. 10.1038/ajg.2012.218. 22890223 PMC3438468

[62] Rahier JF, Magro F, Abreu C. et al. Second European evidence-based consensus on the prevention, diagnosis and management of opportunistic infections in inflammatory bowel disease. *J Crohns Colitis* 2014;8:443–68. 10.1016/j.crohns.2013.12.013. 24613021

[63] Deepak P, Sifuentes H, Sherid M. et al. T-cell non-Hodgkin's lymphomas reported to the FDA AERS with tumor necrosis factor-alpha (TNF-α) inhibitors: results of the REFURBISH study. *Am J Gastroenterol* 2013;108:99–105. 10.1038/ajg.2012.334. 23032984

[64] Farraye FA, Melmed GY, Lichtenstein GR. et al. ACG clinical guideline: preventive care in inflammatory bowel disease. *Am J Gastroenterol* 2017;112:241–58. 28071656 10.1038/ajg.2016.537

[65] Osterman MT, Lichtenstein GR. Current and future anti-TNF therapy for inflammatory bowel disease. *Curr Treat Options Gastroenterol* 2007;10:195–207. 17547858 10.1007/s11938-007-0013-3

[66] Danese S, Semeraro S, Papa A. et al. Extraintestinal manifestations in inflammatory bowel disease. *World J Gastroenterol* 2005;11:7227–36. 16437620 10.3748/wjg.v11.i46.7227 PMC4725142

[67] Pittock SJ, Lennon VA. Aquaporin-4 autoantibodies in a paraneoplastic context. *Arch Neurol* 2008;65:629–32. 18474738 10.1001/archneur.65.5.629

[68] Bloomgren G, Richman S, Hotermans C. et al. Risk of natalizumab-associated progressive multifocal leukoencephalopathy. *N Engl J Med* 2012;366:1870–80. 22591293 10.1056/NEJMoa1107829

[69] Wyant T, Fedyk E, Abhyankar B. An overview of the mechanism of action of the monoclonal antibody vedolizumab. *J Crohns Colitis* 2016;10:1437–44. 27252400 10.1093/ecco-jcc/jjw092

[70] Faleck DM, Winters A, Chablaney S. et al. Shorter disease duration is associated with higher rates of response to vedolizumab in patients with Crohn's disease but not ulcerative colitis. *Clin Gastroenterol Hepatol* 2019;17:2497–2505.e1. 10.1016/j.cgh.2018.12.040. 30625408 PMC7026826

[71] Papp KA, Griffiths CE, Gordon K. et al. Long-term safety of ustekinumab in patients with moderate-to-severe psoriasis: final results from 5 years of follow-up. *Br J Dermatol* 2013;168:844–54. 10.1111/bjd.12214. 23301632

[72] Ryan C, Menter A, Guenther L. et al. Efficacy and safety of ustekinumab over 4 years in patients with moderate-to-severe psoriasis: results from the PHOENIX 1 and PHOENIX 2 trials. *J Am Acad Dermatol* 2015;173:1514–22.

[73] Ghosh S, Panaccione R. Anti-adhesion molecule therapy for inflammatory bowel disease. *Ther Adv Gastroenterol* 2010;3:239–58. 21180606 10.1177/1756283X10373176 PMC3002582

[74] Burisch J, Jess T, Martinato M. et al. The burden of inflammatory bowel disease in Europe. *J Crohns Colitis* 2013;7:322–37. 10.1016/j.crohns.2013.01.010. 23395397

[75] Danese S, Gomollon F, Governing Board and Operational Board of ECCO. ECCO position statement: the use of biosimilar medicines in the treatment of inflammatory bowel disease (IBD). *J Crohns Colitis* 2013;7:586–9. 10.1016/j.crohns.2013.03.011. 23623738

[76] Jorgensen KK, Olsen IC, Goll GL. et al. Switching from originator infliximab to biosimilar CT-P13 compared with maintained treatment with originator infliximab (NOR-SWITCH): a 52-week, randomised, double-blind, non-inferiority trial. *Lancet.* 2017;389:2304–16. 10.1016/S0140-6736(17)30068-5. 28502609

[77] Bodger K, Kikuchi T, Hughes D. Cost-effectiveness of biological therapy for Crohn's disease: Markov cohort analyses incorporating United Kingdom patient-level cost data. *Aliment Pharmacol Ther* 2009;30:265–74. 10.1111/j.1365-2036.2009.04033.x. 19438428

[78] Goll GL, Jorgensen KK, Sexton J. et al. Long-term efficacy and safety of biosimilar infliximab (CT-P13) after switching from originator infliximab: open-label extension of the NOR-SWITCH trial. *J Intern Med* 2019;285:653–69. 10.1111/joim.12880. 30762274 PMC6850326

[79] Glintborg B, Sørensen IJ, Loft AG. et al. A nationwide non-medical switch from originator infliximab to biosimilar CT-P13 in 802 patients with inflammatory arthritis: 1-year clinical outcomes from the DANBIO registry. *Ann Rheum Dis* 2017;76:1426–31. 10.1136/annrheumdis-2016-210742. 28473425

[80] Mulcahy AW, Hlavka JP, Case SR. Biosimilar cost savings in the United States: initial experience and future potential. *Rand Health Q* 2018;7:3. 10.7249/rhq-07-04-03 PMC6075809 30083415

[81] Yazdany J, Dudley RA, Chen R. et al. Coverage for high-cost specialty drugs for rheumatoid arthritis in Medicare part *D*. *Arthritis Rheumatol* 2015;67:1474–80. 25900105 10.1002/art.39079 PMC4464809

[82] IQVIA Institute for Human Data Science . Biosimilars in the United States 2023–2027: Competition, Savings, and Sustainability. USA: IQVIA Institute, 2023.

[83] Weeda ER, Tkacz J, Piercy J. et al. Impact of prior authorization on treatment discontinuation among patients with rheumatoid arthritis. *Am J Manag Care* 2018;24:SP282–8.

[84] Wentworth BJ, Buerlein RCD, Tuskey AG. et al. Patients' perspectives on barriers to medication adherence in inflammatory bowel disease: a qualitative assessment. *Inflamm Bowel Dis* 2018;24:1900–7.

[85] Kotze PG, Underwood FE, Damião AOMC. et al. Progression of inflammatory bowel diseases throughout Latin America and the Caribbean: a systematic review. *Clin Gastroenterol Hepatol* 2020;18:304–12. 10.1016/j.cgh.2019.06.030. 31252191

[86] Yamamoto-Furusho JK, Parra-Holguín NN, Juliao-Baños F. et al. Diagnosis and treatment of inflammatory bowel disease: first Latin American consensus of the pan American Crohn's and colitis organisation. *Rev Gastroenterol Mex* 2017;82:46–84. 27979414 10.1016/j.rgmx.2016.07.003

[87] Dudkowiak R, Neubauer K, Poniewierka E. Biosimilars in the treatment of inflammatory bowel diseases. *Prz Gastroenterol* 2018;13:85–92.30002765

[88] Alatab S, Sepanlou SG, Ikuta K. et al. The global, regional, and national burden of inflammatory bowel disease in 195 countries and territories, 1990-2017: a systematic analysis for the global burden of disease study 2017. *Lancet Gastroenterol Hepatol* 2020;5:17–30. 31648971 10.1016/S2468-1253(19)30333-4 PMC7026709

[89] Ahuja V, Tandon RK. Inflammatory bowel disease in the Asia-Pacific area: a comparison with developed countries and regional differences. *J Dig Dis* 2010;11:134–47. 10.1111/j.1751-2980.2010.00429.x. 20579217

[90] National Institute for Health and Care Excellence . Vedolizumab for Treating Moderately to Severely Active Crohn's Disease after Prior Therapy. Technology Appraisal Guidance [TA352]. New Zealand: Pharmacoeconomics, 2023.

[91] Marshall JK, Bessette LG, Thorne JC. et al. Cost-effectiveness of risankizumab versus ustekinumab for the treatment of moderate-to-severe Crohn's disease in Canada. *J Med Econ* 2023;26:432–43.

[92] Siegel CA . Shared decision making in inflammatory bowel disease: helping patients understand the tradeoffs between treatment options. *Gut.* 2012;61:459–65. 22187072 10.1136/gutjnl-2011-300988

[93] Vavricka SR, Bentele N, Scharl M. et al. Systematic evaluation of risk factors for diagnostic delay in inflammatory bowel disease. *Inflamm Bowel Dis* 2012;18:496–505. 21509908 10.1002/ibd.21719

[94] Schreiber S, Dignass A, Peyrin-Biroulet L. et al. Systematic review with meta-analysis: real-world effectiveness and safety of vedolizumab in patients with inflammatory bowel disease. *J Gastroenterol* 2018;53:1048–64. 10.1007/s00535-018-1480-0. 29869016 PMC6132930

[95] Ghosh S, Sanchez Gonzalez Y, Zhou W. et al. Ustekinumab treatment and dosing patterns in patients with moderately-to-severely active ulcerative colitis in routine practice in the United States. *Curr Med Res Opin* 2020;36:875–83.31990207

[96] Kane SV, Cohen RD, Aikens JE. et al. Prevalence of nonadherence with maintenance mesalamine in quiescent ulcerative colitis. *Am J Gastroenterol* 2001;96:2929–33. 11693328 10.1111/j.1572-0241.2001.04683.x

[97] Selinger CP, Robinson A, Leong RW. Clinical impact and drivers of non-adherence to maintenance medication for inflammatory bowel disease. *Expert Opin Drug Saf* 2011;10:863–70. 10.1517/14740338.2011.583915. 21548837

[98] Waters BM, Jensen L, Fedorak RN. Effects of formal education for patients with inflammatory bowel disease: a randomized controlled trial. *Can J Gastroenterol* 2005;19:235–44. 15861266 10.1155/2005/250504

[99] Con D, De Cruz P. Mobile phone apps for inflammatory bowel disease self-management: a systematic assessment of content and tools. *JMIR Mhealth Uhealth* 2016;4:e13, p.13. 10.2196/mhealth.4874. 26831935 PMC4754530

[100] Cross RK, Cheevers N, Rustgi A. et al. Randomized, controlled trial of home telemanagement in patients with ulcerative colitis (UC HAT). *Inflamm Bowel Dis* 2012;18:1018–25. 10.1002/ibd.21795. 21688350 PMC3179574

[101] Siegel CA, Levy LC, Mackenzie TA. et al. Patient perceptions of the risks and benefits of infliximab for the treatment of inflammatory bowel disease. *Inflamm Bowel Dis* 2008;14:1–6. 17924559 10.1002/ibd.20283

[102] Strehl C, Bijlsma JW, de Wit M. et al. Defining conditions where long-term glucocorticoid treatment has an acceptably low level of harm to facilitate implementation of existing recommendations: viewpoints from an EULAR task force. *Ann Rheum Dis* 2016;75:952–7. 26933146 10.1136/annrheumdis-2015-208916

[103] Kane S, Huo D, Aikens J. et al. Medication nonadherence and the outcomes of patients with quiescent ulcerative colitis. *Am J Med* 2003;114:39–43. 12543288 10.1016/s0002-9343(02)01383-9

[104] Jackson CA, Clatworthy J, Robinson A. et al. Factors associated with non-adherence to oral medication for inflammatory bowel disease: a systematic review. *Am J Gastroenterol* 2010;105:525–39. 10.1038/ajg.2009.685. 19997092

[105] Biasci D, Lee JC, Noor NM. et al. A blood-based prognostic biomarker in IBD. *Gut.* 2019;68:1386–95. 31030191 10.1136/gutjnl-2019-318343 PMC6691955

[106] Steenholdt C, Palarasah Y, Bendtzen K. et al. Pre-existing IgG antibodies cross-reacting with the fab region of infliximab predict efficacy and safety of infliximab therapy in inflammatory bowel disease. *Aliment Pharmacol Ther* 2013;37:1172–83. 23650912 10.1111/apt.12330

[107] Kirchgesner J, Lemaitre M, Carrat F. et al. Risk of serious and opportunistic infections associated with treatment of inflammatory bowel diseases. *Gastroenterology.* 2018;155:337–346.e10. 10.1053/j.gastro.2018.04.012. 29655835

[108] Ye BD, Pesegova M, Alexeeva O. et al. Efficacy and safety of biosimilar CT-P13 compared with originator infliximab in patients with active Crohn's disease: an international, randomised, double-blind, phase 3 non-inferiority study. *Lancet.* 2019;393:1699–707. 30929895 10.1016/S0140-6736(18)32196-2

[109] Ngo VL, Abo H, Maxim E. et al. A cytokine network involving IL-36γ, IL-23, and IL-22 promotes antimicrobial defense and recovery from intestinal barrier damage. *Proc Natl Acad Sci USA* 2018;115:E5076–85. 10.1073/pnas.1718902115. 29760082 PMC5984499

[110] Nishida A, Hidaka K, Kanda T. et al. Increased expression of interleukin-36, a member of the interleukin-1 cytokine family, in inflammatory bowel disease. *Inflamm Bowel Dis* 2016;22:303–14. 26752465 10.1097/MIB.0000000000000654

[111] Khoury T, Tzur D, Margalit S. et al. The IL-36 pathway in inflammatory bowel disease. *J Crohns Colitis* 2020;14:1253–61.

[112] Scheibe K, Kersten C, Schmied A. et al. Inhibiting interleukin 36 receptor signaling reduces fibrosis in mice with chronic intestinal inflammation. *Gastroenterology.* 2019;156:1082–97. 30452921 10.1053/j.gastro.2018.11.029

[113] Boutet MA, Najm A, Bart G. et al. IL-38 overexpression induces anti-inflammatory effects in mice arthritis models and in human macrophages in vitro. *Ann Rheum Dis* 2017;76:1304–12. 28288964 10.1136/annrheumdis-2016-210630

[114] Reich K, Papp KA, Blauvelt A. et al. Spesolimab for the treatment of generalized pustular psoriasis. *N Engl J Med* 2021;385:2431–40. 34936739 10.1056/NEJMoa2111563

[115] Harbour SN, Maynard CL, Zindl CL. et al. Th17 cells give rise to Th1 cells that are required for the pathogenesis of colitis. *Proc Natl Acad Sci USA* 2015;112:7061–6. 10.1073/pnas.1415675112. 26038559 PMC4460486

[116] Lee JS, Tato CM, Joyce-Shaikh B. et al. Interleukin-23-independent IL-17 production regulates intestinal epithelial permeability. *Immunity.* 2015;43:727–38. 10.1016/j.immuni.2015.09.003. 26431948 PMC6044435

[117] Hueber W, Sands BE, Lewitzky S. et al. Secukinumab, a human anti-IL-17A monoclonal antibody, for moderate to severe Crohn's disease: unexpected results of a randomised, double-blind placebo-controlled trial. *Gut.* 2012;61:1693–700. 10.1136/gutjnl-2011-301668. 22595313 PMC4902107

[118] Ramirez-Carrozzi V, Sambandam A, Luis E. et al. IL-17C regulates the innate immune function of epithelial cells in an autocrine manner. *Nat Immunol* 2011;12:1159–66. 10.1038/ni.2156. 21993848

[119] Song X, Zhu S, Shi P. et al. IL-17RE is the functional receptor for IL-17C and mediates mucosal immunity to infection with intestinal pathogens. *Nat Immunol* 2011;12:1151–8. 10.1038/ni.2155. 21993849

[120] Maxwell JR, Zhang Y, Brown WA. et al. Differential roles for interleukin-23 and interleukin-17 in intestinal immunoregulation. *Immunity.* 2015;43:739–50. 26431947 10.1016/j.immuni.2015.08.019

[121] Monteleone G, Neurath MF, Ardizzone S. et al. Mongersen, an oral SMAD7 antisense oligonucleotide, and Crohn's disease. *N Engl J Med* 2015;372:1104–13. 10.1056/NEJMoa1407250. 25785968

[122] Monteleone G, Kumberova A, Croft NM. et al. Blocking Smad7 restores TGF-beta1 signaling in chronic inflammatory bowel disease. *J Clin Invest* 2001;108:601–9. 11518734 10.1172/JCI12821 PMC209401

[123] Feagan BG, Sands BE, Rossiter G. et al. Effects of mongersen (GED-0301) on endoscopic and clinical outcomes in patients with active Crohn's disease. *Gastroenterology.* 2018;154:61–64.e6. 10.1053/j.gastro.2017.08.035. 28847751

[124] Boirivant M, Pallone F, Di Giacinto C. et al. Inhibition of Smad7 with a specific antisense oligonucleotide facilitates TGF-beta1-mediated suppression of colitis. *Gastroenterology.* 2006;131:1786–98. 17087939 10.1053/j.gastro.2006.09.016

[125] Rieder F, Zimmermann EM, Remzi FH. et al. Crohn's disease complicated by strictures: a systematic review. *Gut.* 2013;62:1072–84. 10.1136/gutjnl-2012-304353. 23626373 PMC4884453

[126] Ning C, Wang X, Gao S. et al. Complement activation contributes to peritoneal fibrosis through the C5a-C5aR1 axis. *Lab Investig* 2018;98:1313–24.

[127] Kotwal GJ, Moss B. Analysis of a large cluster of proteins involved in yaba-like disease virus pathogenesis. *Virology.* 1988;167:524–37. 2849238

[128] Cleynen I, Vazeille E, Artieda M. et al. Genetic evidence supporting the association of protease and protease inhibitor genes with inflammatory bowel disease: a systematic review. *PLoS ONE* 2011;6:e24106. 10.1371/journal.pone.0024106. 21931648 PMC3169567

[129] Ricklin D, Hajishengallis G, Yang K. et al. Complement: a key system for immune surveillance and homeostasis. *Nat Immunol* 2010;11:785–97. 10.1038/ni.1923. 20720586 PMC2924908

[130] Hillmen P, Young NS, Schubert J. et al. The complement inhibitor eculizumab in paroxysmal nocturnal hemoglobinuria. *N Engl J Med* 2006;355:1233–43. 16990386 10.1056/NEJMoa061648

[131] Noris M, Remuzzi G. Overview of complement activation and regulation. *Semin Nephrol* 2013;33:479–92. 10.1016/j.semnephrol.2013.08.001. 24161035 PMC3820029

[132] Gevers D, Kugathasan S, Denson LA. et al. The treatment-naive microbiome in new-onset Crohn's disease. *Cell Host Microbe* 2014;15:382–92. 10.1016/j.chom.2014.02.005. 24629344 PMC4059512

[133] Palmela C, Chevarin C, Xu Z. et al. Adherent-invasive Escherichia coli in inflammatory bowel disease. *Gut.* 2018;67:574–87. 10.1136/gutjnl-2017-314903. 29141957

[134] Lodes MJ, Cong Y, Elson CO. et al. Bacterial flagellin is a dominant antigen in Crohn disease. *J Clin Invest* 2004;113:1296–306. 15124021 10.1172/JCI20295 PMC398429

[135] Targan SR, Landers CJ, Yang H. et al. Antibodies to CBir1 flagellin define a unique response that is associated independently with complicated Crohn's disease. *Gastroenterology.* 2005;128:2020–8. 15940634 10.1053/j.gastro.2005.03.046

[136] Sitaraman SV, Klapproth JM, Moore DA 3rd. et al. Elevated flagellin-specific immunoglobulins in Crohn's disease. *Am J Physiol Gastrointest Liver Physiol* 2005;288:G403–6. 10.1152/ajpgi.00357.2004. 15388489

[137] Chassaing B, Darfeuille-Michaud A. The commensal microbiota and enteropathogens in the pathogenesis of inflammatory bowel diseases. *Gastroenterology.* 2011;140:1720–8. 21530738 10.1053/j.gastro.2011.01.054

[138] Cario E, Rosenberg IM, Brandwein SL. et al. Lipopolysaccharide activates distinct signaling pathways in intestinal epithelial cell lines expressing toll-like receptors. *J Immunol* 2000;164:966–72. 10623846 10.4049/jimmunol.164.2.966

[139] Atarashi K, Tanoue T, Shima T. et al. Induction of colonic regulatory T cells by indigenous clostridium species. *Science.* 2011;331:337–41. 10.1126/science.1198469. 21205640 PMC3969237

[140] Shih DQ, Zheng L, Zhang X. et al. Inhibition of a novel fibrogenic factor Tl1a reverses established colonic fibrosis. *Mucosal Immunol* 2014;7:1492–503. 10.1038/mi.2014.37. 24850426 PMC4205266

[141] Bamias G, Siakavellas SI, Stamatelopoulos KS. et al. Circulating levels of TNF-like cytokine 1A (TL1A) and its decoy receptor 3 (DcR3) in rheumatoid arthritis. *Clin Immunol* 2008;129:249–55. 10.1016/j.clim.2008.07.014. 18757243

[142] Danese S, Klopocka M, Scherl EJ. et al. Anti-TL1A antibody PF-06480605 safety and efficacy for ulcerative colitis: a phase 2a single-arm study. *Clin Gastroenterol Hepatol* 2021;19:2324–2332.e6. 10.1016/j.cgh.2021.06.011. 34126262

[143] Takedatsu H, Michelsen KS, Wei B. et al. TL1A (TNFSF15) regulates the development of chronic colitis by modulating both T-helper 1 and T-helper 17 activation. *Gastroenterology.* 2008;135:552–67. 10.1053/j.gastro.2008.04.037. 18598698 PMC2605110

[144] Shih DQ, Targan SR. Insights into IBD pathogenesis. *Curr Gastroenterol Rep* 2009;11:473–80. 19903423 10.1007/s11894-009-0072-9 PMC2895981

[145] Kontermann RE, Brinkmann U. Bispecific antibodies. *Drug Discov Today* 2015;20:838–47. 10.1016/j.drudis.2015.02.008. 25728220

[146] Brinkmann U, Kontermann RE. The making of bispecific antibodies. *MAbs.* 2017;9:182–212. 28071970 10.1080/19420862.2016.1268307 PMC5297537

[147] Sedykh SE, Prinz VV, Buneva VN. et al. Bispecific antibodies: design, therapy, perspectives. *Drug Des Devel Ther* 2018;12:195–208. 10.2147/DDDT.S151282. PMC5784585 29403265

[148] Genovese MC, Greenwald M, Codding C. et al. A phase 2 randomized study of subcutaneous ixekizumab, an anti-interleukin-17 monoclonal antibody, in rheumatoid arthritis patients who were naive to biologic agents or had an inadequate response to tumor necrosis factor inhibitors. *Arthritis Rheumatol* 2014;66:1693–704. 24623718 10.1002/art.38617

[149] Ito R, Shin-Ya M, Kishida T. et al. Interferon-gamma is causatively involved in experimental inflammatory bowel disease in mice. *Clin Exp Immunol* 2006;146:330–8. 17034586 10.1111/j.1365-2249.2006.03214.x PMC1942055

[150] DiCioccio AT, Wilkins JJ, Novoszel P. et al. Interplay between a dual variable domain immunoglobulin (DVD-Ig) and low-molecular-weight inhibitors: demonstration of proof-of-concept in a rheumatoid arthritis model. *Clin Exp Rheumatol* 2015;33:351–9.

[151] Neurath MF . IL-23 in inflammatory bowel diseases and colon cancer. *Cytokine Growth Factor Rev* 2019;45:1–8. 10.1016/j.cytogfr.2018.12.002. 30563755

[152] Fuchs F, Schillinger U, Atreya R. et al. Clinical, endoscopic, transmural, and histologic healing effects of PTG-100, an anti-alpha4beta7/anti-TNFalpha bispecific antibody, in the chronic DSS colitis model. *Gastroenterology.* 2017;152:S599.

[153] Blat D, Zigmond E, Alteber Z. et al. Cell-selective targeting of anti-CEA/anti-CD3 bispecific antibody for the treatment of colorectal cancer. *Oncotarget.* 2017;8:14992–5004.

[154] Deschoolmeester V, Baay M, Van Marck E. et al. Tumor infiltrating lymphocytes: an intriguing player in the survival of colorectal cancer patients. *BMC Immunol* 2010;11:19. 20385003 10.1186/1471-2172-11-19 PMC2864219

[155] Klein C, Schaefer W, Regula JT. The use of CrossMAb technology for the generation of bi- and multispecific antibodies. *MAbs.* 2016;8:1010–20. 10.1080/19420862.2016.1197457. 27285945 PMC4968094

[156] Beck A, Goetsch L, Dumontet C. et al. Strategies and challenges for the next generation of antibody-drug conjugates. *Nat Rev Drug Discov* 2017;16:315–37. 10.1038/nrd.2016.268. 28303026

[157] Drago JZ, Modi S, Chandarlapaty S. Unlocking the potential of antibody-drug conjugates for cancer therapy. *Nat Rev Clin Oncol* 2021;18:327–44. 33558752 10.1038/s41571-021-00470-8 PMC8287784

[158] Chalouni C, Doll S. Fate of antibody-drug conjugates in cancer cells. *J Exp Clin Cancer Res* 2018;37:20. 10.1186/s13046-017-0667-1. 29409507 PMC5802061

[159] Kugathasan S, Denson LA, Walters TD. et al. Prediction of complicated disease course for children newly diagnosed with Crohn's disease: a multicentre inception cohort study. *Lancet.* 2017;389:1710–8. 10.1016/S0140-6736(17)30317-3. 28259484 PMC5719489

[160] Haritunians T, Taylor KD, Targan SR. et al. Genetic predictors of medically refractory ulcerative colitis. *Inflamm Bowel Dis* 2010;16:1830–40. 20848476 10.1002/ibd.21293 PMC2959149

[161] Biscaglia F, Rajendran S, Conflitti P. et al. Artificial intelligence and inflammatory bowel disease: where are we going? *World J Gastroenterol* 2021;27:6295–315.

[162] Viennois E, Chassaing B. First victim, later witness: microbiota-derived extracellular vesicles in intestinal inflammation. *Nat Rev Gastroenterol Hepatol* 2018;15:387–8.29703976

[163] Ananthakrishnan AN, Luo C, Yajnik V. et al. Gut microbiome function predicts response to anti-integrin biologic therapy in inflammatory bowel diseases. *Cell Host Microbe* 2017;21:603–610.e3. 10.1016/j.chom.2017.04.010. 28494241 PMC5705050

[164] Vashist SK, Luong JHT. Point-of-care technologies enabling next-generation healthcare monitoring and management. *Biotechnol Adv* 2017;35:153–76.

[165] Danese S, Fiocchi C. Etiopathogenesis of inflammatory bowel diseases. *World J Gastroenterol* 2006;12:4807–12. 16937461 10.3748/wjg.v12.i30.4807 PMC4087613

[166] Verstockt B, Ferrante M, Vermeire S. et al. New treatment options for inflammatory bowel diseases. *J Gastroenterol* 2018;53:585–90. 10.1007/s00535-018-1449-z. 29556726 PMC5910475

[167] Sandborn WJ, Ghosh S, Panes J. et al. Tofacitinib, an oral Janus kinase inhibitor, in active ulcerative colitis. *N Engl J Med* 2012;367:616–24. 10.1056/NEJMoa1112168. 22894574

[168] Neurath MF . Targeting immune cell circuits and trafficking in inflammatory bowel disease. *Nat Immunol* 2019;20:970–9. 10.1038/s41590-019-0415-0. 31235952

[169] Yao Y, Xu X, Zhang G. et al. Role of HDAC2 in the anti-inflammatory effects of budesonide in a mouse model of asthma. *J Immunol* 2013;191:4750–9.

[170] Zhang L, Gu FX, Chan JM. et al. Nanoparticles in medicine: therapeutic applications and developments. *Clin Pharmacol Ther* 2008;83:761–9. 17957183 10.1038/sj.clpt.6100400

[171] Pridgen EM, Alexis F, Kuo TT. et al. Transepithelial transport of fc-targeted nanoparticles by the neonatal fc receptor for oral delivery. *Sci Transl Med* 2013;5:167–173. 10.1126/scitranslmed.3007049. PMC4023672 24285486

[172] Moroz E, Matoori S, Leroux JC. Oral delivery of macromolecular drugs: where we are after almost 100 years of attempts. *Adv Drug Deliv Rev* 2016;101:108–21. 10.1016/j.addr.2016.01.010. 26826437

[173] Renukuntla J, Vadlapudi AD, Patel A. et al. Approaches for enhancing oral bioavailability of peptides and proteins. *Int J Pharm* 2013;447:75–93. 10.1016/j.ijpharm.2013.02.030. 23428883 PMC3680128

[174] Goldberg M, Gomez-Orellana I. Challenges for the oral delivery of macromolecules. *Nat Rev Drug Discov* 2003;2:289–95. 12669028 10.1038/nrd1067

[175] Singh S, Murad MH, Fumery M. et al. Comparative efficacy and safety of biologic therapies for moderate-to-severe Crohn's disease: a systematic review and network meta-analysis. *Lancet Gastroenterol Hepatol* 2021;6:1002–14. 34688373 10.1016/S2468-1253(21)00312-5 PMC8933137

[176] Alric H, Amiot A, Kirchgesner J. et al. The effectiveness of either ustekinumab or vedolizumab in 239 patients with Crohn's disease refractory to anti-tumour necrosis factor. *Aliment Pharmacol Ther* 2020;51:948–57. 10.1111/apt.15706. 32249966

[177] Lujan R, Mañosa M, Gordillo J. et al. Comparative effectiveness of ustekinumab and adalimumab in anti-TNF-naïve Crohn's disease patients: results from the ENEIDA registry. *J Crohns Colitis* 2024;18:512–21.

[178] Yiu ZZN, Warren RB, Mrowietz U. et al. Safety of biologics in psoriasis: a network meta-analysis of clinical trial data. *J Invest Dermatol* 2022;142:638–47.

[179] Solitano V, Facciorusso A, Jess T. et al. Comparative risk of serious infections with biologic agents and oral small molecules in inflammatory bowel diseases: a systematic review and meta-analysis. *Clin Gastroenterol Hepatol* 2023;21:907–21. 35944832 10.1016/j.cgh.2022.07.032

[180] Present DH, Rutgeerts P, Targan S. et al. Infliximab for the treatment of fistulas in patients with Crohn's disease. *N Engl J Med* 1999;340:1398–405. 10228190 10.1056/NEJM199905063401804

[181] Turner D, Ricciuto A, Lewis A. et al. STRIDE-II: an update on the selecting therapeutic targets in inflammatory bowel disease (STRIDE) initiative of the International Organization for the Study of IBD (IOIBD): determining therapeutic goals for treat-to-target strategies in IBD. *Gastroenterology.* 2021;160:1570–83. 10.1053/j.gastro.2020.12.031. 33359090

[182] Danese S, Vermeire S, D'Haens G. et al. Treat to target versus standard of care for patients with Crohn's disease treated with ustekinumab (STARDUST): an open-label, multicentre, randomised phase 3b trial. *Lancet Gastroenterol Hepatol* 2022;7:294–306. 35120656 10.1016/S2468-1253(21)00474-X

[183] Liu Y, DiStasio M, Su G. et al. High-plex protein and whole transcriptome co-mapping at cellular resolution with spatial CITE-seq. *Nat Biotechnol* 2023;41:1405–9. 10.1038/s41587-023-01676-0. 36823353 PMC10567548

[184] Fan R, Zhang D, Rodríguez-Kirby L. et al. Spatial dynamics of mammalian brain development and neuroinflammation by multimodal tri-omics mapping. *Res Sq [Preprint]* 2024;rs.3.rs-4814866. 10.21203/rs.3.rs-4814866/v1.

[185] Fan R, Baysoy A, Tian X. et al. Spatially resolved panoramic in vivo CRISPR screen via perturb-DBiT. *Res Sq [Preprint]* 2025;rs.3.rs-6481967. 10.21203/rs.3.rs-6481967/v1.

